# Supplementary material for: Tumor-specific cytosol-penetrating antibodies for antigen- and TME-dependent intracellular cargo delivery
Source: Mol Ther Oncol. 2025 Jan 2;33(1):200931. doi: 10.1016/j.omton.2024.200931 (PMC11786873; doi:10.1016/j.omton.2024.200931)
Supplement: Document S1. Figures S1–S14 [file mmc1.pdf]

OMTON, Volume *33*

Supplemental information

Tumor-specific cytosol-penetrating antibodies for antigen- and TME-dependent intracellular cargo delivery

Carolin Sophie Dombrowsky, Felix Klaus Geyer, Diana Zakharchuk, and Harald Kolmar

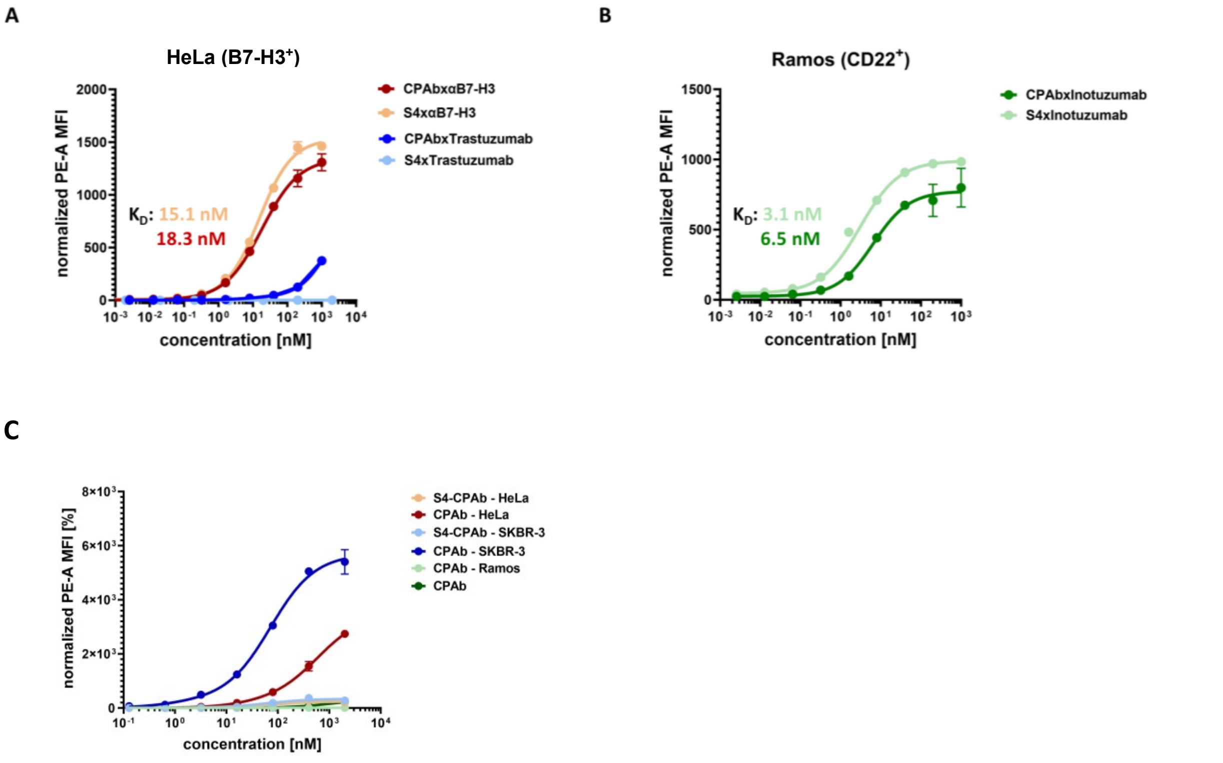

Figure S1. On-cell binding assay of the masked and unmasked bispecific antibodies on Hela and Ramos cells respectively. The cells were treated with concentrations of the compound ranging from 0.002 nM to 2000 nM. The K_D values were calculated from variable slope four-parameter fitting using GraphPad Prism 10.1.0 (316) and presented in the corresponding graph. Comparative analysis for the investigation of HSPG-binding impact on the on-cell binding were conducted in (A) HeLa cells with masked S4xαB7-H3, unmasked CPAbxαB7-H3, and masked and unmasked isotype controls, in (B) Ramos cells with masked and unmasked S4xInotuzumab, and in (C) previously published masked S4-CPAb and unmasked CPAb.

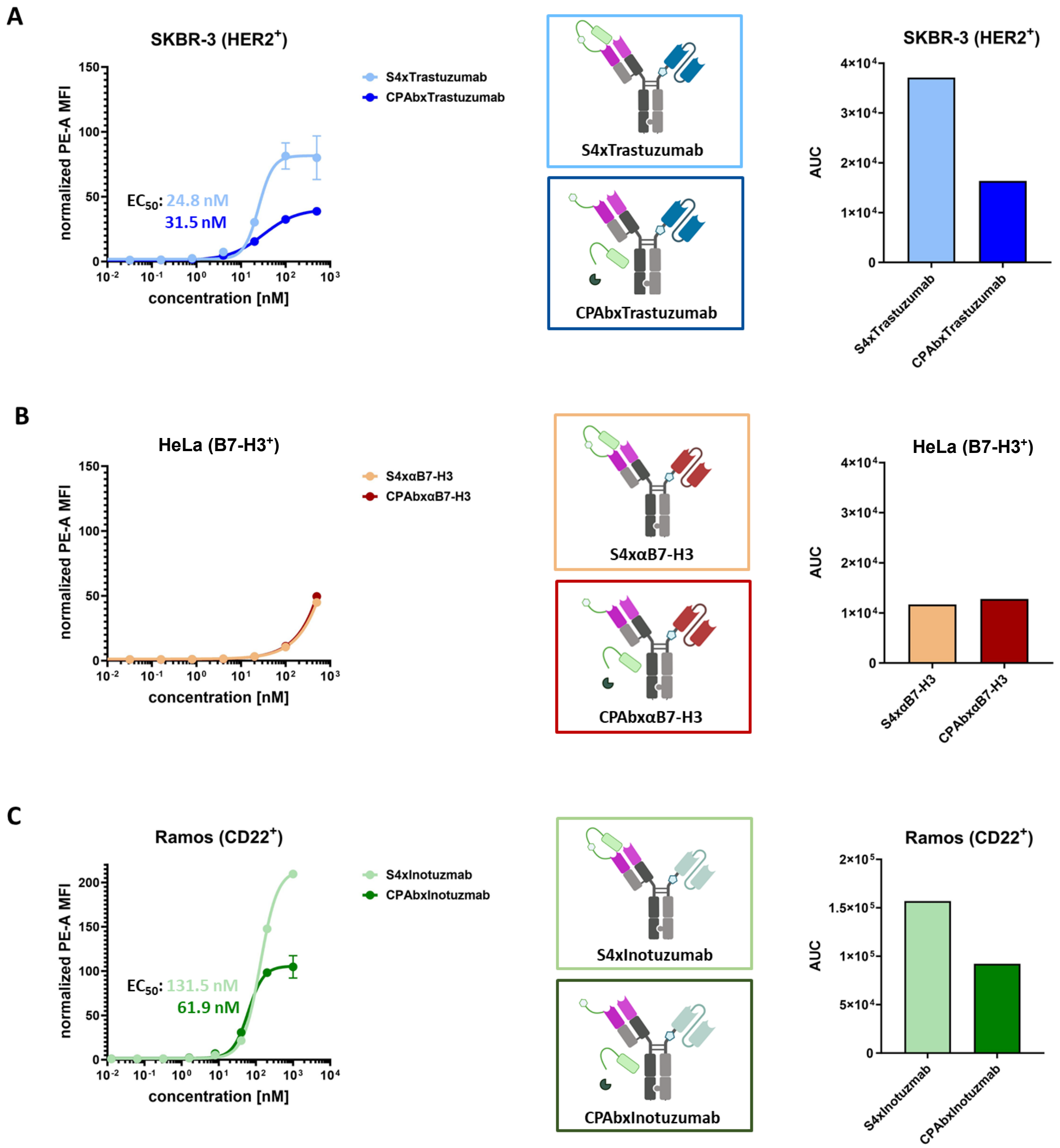

Figure S2. Determination of the internalization of the masked and unmasked bispecific constructs in comparison. The respective cell lines (a) SKBR-3, (b) HeLa, and (c) Ramos were incubated with the antibody-dye conjugates (DAR: S4/CPAbxInotuzumab: 8.9; S4/CPAbxTrastuzumab: 4.6; S4/CPAbxαB7-H3: 8.5). The visualization of the area under the curve of the constructs allows for a comparison of the amount of internalizing antibodies. The resulting data points, depicted as mean and error bars, represent the standard deviation derived from experimental duplicates. The EC_{50} values were determined from a four-parameter variable slope fit using GraphPad Prism 10.1.0 (316).

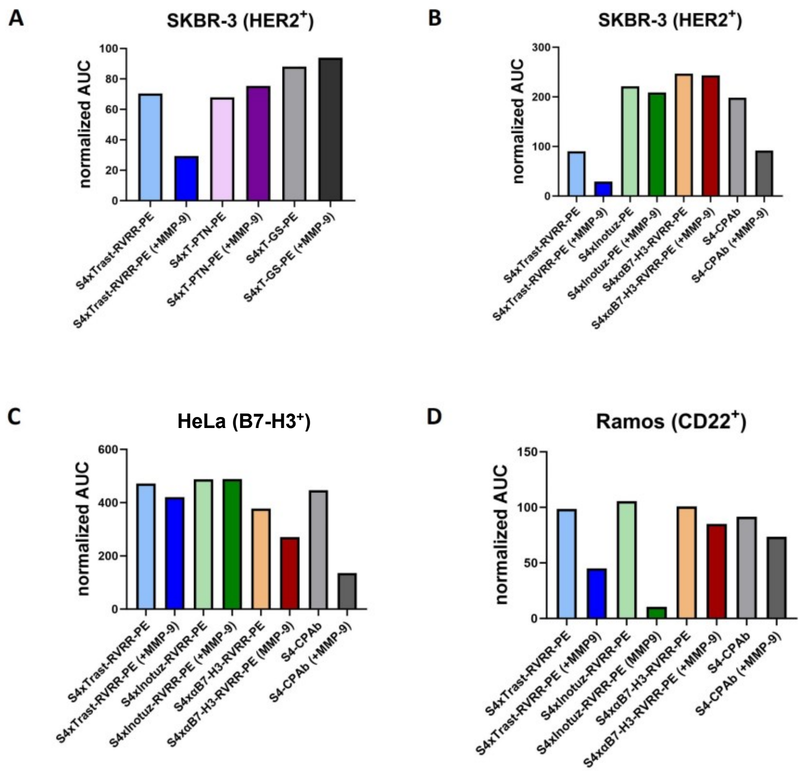

Figure S3. Comparison of linker-dependent and cell-specific cytosolic penetration of bivalent or bispecific constructs in SKBR-3, HeLa and Ramos cells via AUC determination. The visualization of the area under the curve normalized to the upper plateau of the constructs allows for a comparison besides calculated EC_{50} values. The AUCs were determined using GraphPad Prism 10.1.0 (316). (A) Comparison of AUCs of S4xTrastuzumab-RVRR-PE_{cat} (MMP-9 cleaved and untreated), masked and unmasked S4xTrastuzumab-GS-PE_{cat} (non-cleavable) and S4xTrastuzumab-PTN-PE_{cat} (Legumain cleavable) in SKBR-3 cells. AUCs of S4-CPAb, S4xTrastuzumab-RVRR-PE, S4xInotuzumab-RVRR-PE, and S4xαB7-H3-RVRR-PE were compared in (B) SKBR-3, (C) HeLa, and (D) Ramos cells.

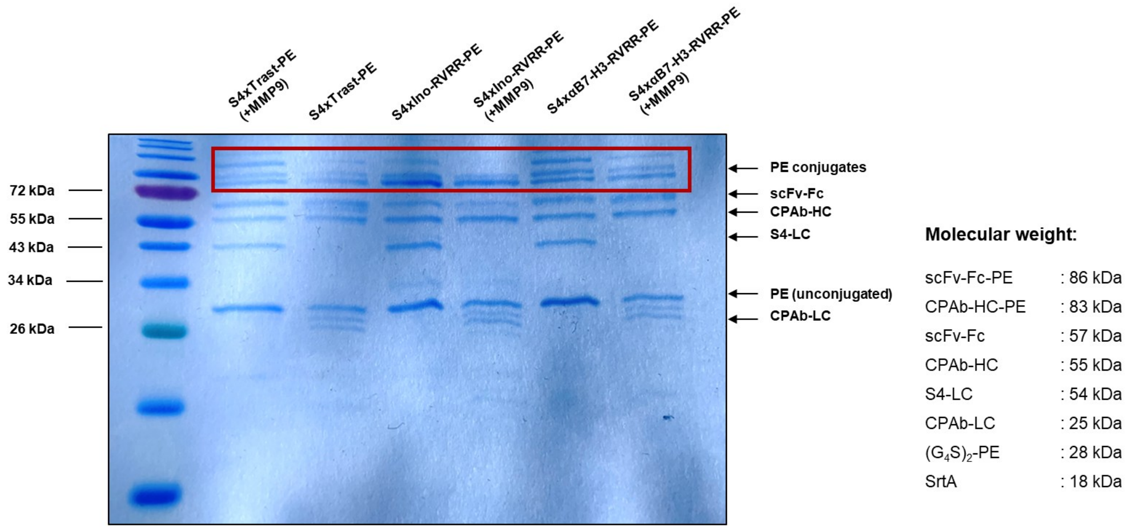

Figure S4. SDS-Gel of antibody-PE_{cat} conjugates. 1.5 µg of S4xTrastuzumab (MMP-9 cleaved and uncleaved), S4xInotuzumab (MMP-9 cleaved and uncleaved), and S4xαB7-H3 (MMP-9 cleaved and uncleaved) were loaded on a reducing SDS-gel. Color Prestained Protein Standard, Broad Range (10–250 kDa) (NEB) was utilized as marker. The red frame highlights the PE_{cat}-coupled heavy chains.

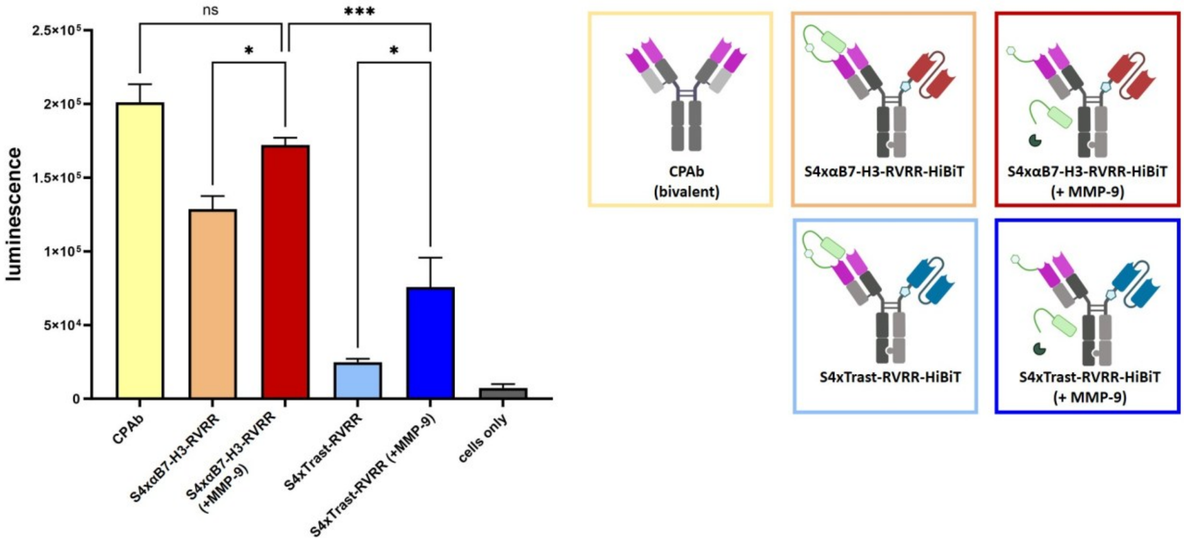

Figure S5. NanoBiT assay analyzing S4xαB7-H3-RVRR-HiBiT variants in comparison with CPAb-HiBiT and S4xTrastuzumab-RVRR-HiBiT in HeLa 11ht LgBiT cells. The results from experimental duplicates were shown as mean values with error bars representing the standard deviation. An unpaired two-tailed t-test (with P value style GP: 0.1234 (ns), 0.0332 (*), and 0.0021 (**)) was utilized to display the significance level (with definition of statistical significance: $P < 0.05$) using GraphPad Prism 10.1.0 (316).

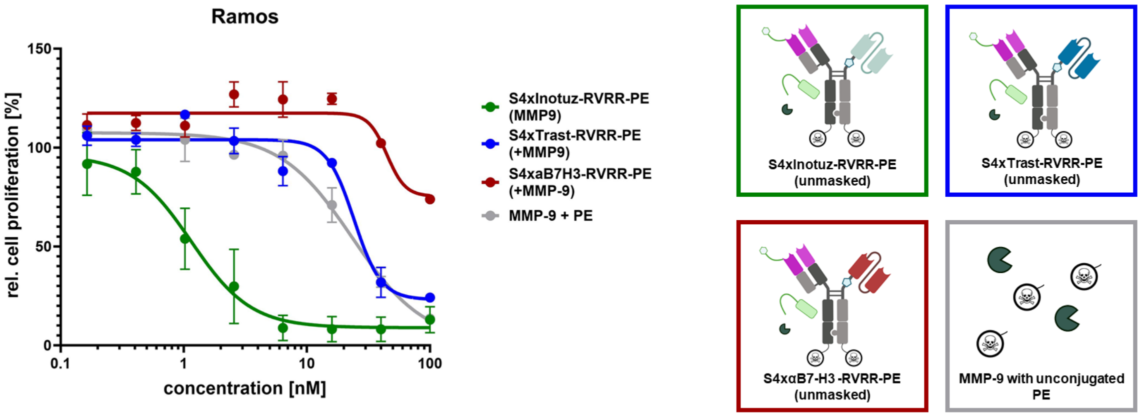

Figure S6. PE-mediated proliferation of MMP-9 cleaved bispecific constructs in Ramos cells in comparison to MMP-9 with free PE_{cat}. The resulting data points are shown as mean and error bars that represent standard deviation derived from experimental duplicates and illustrated using GraphPad Prism 10.1.0 (316).

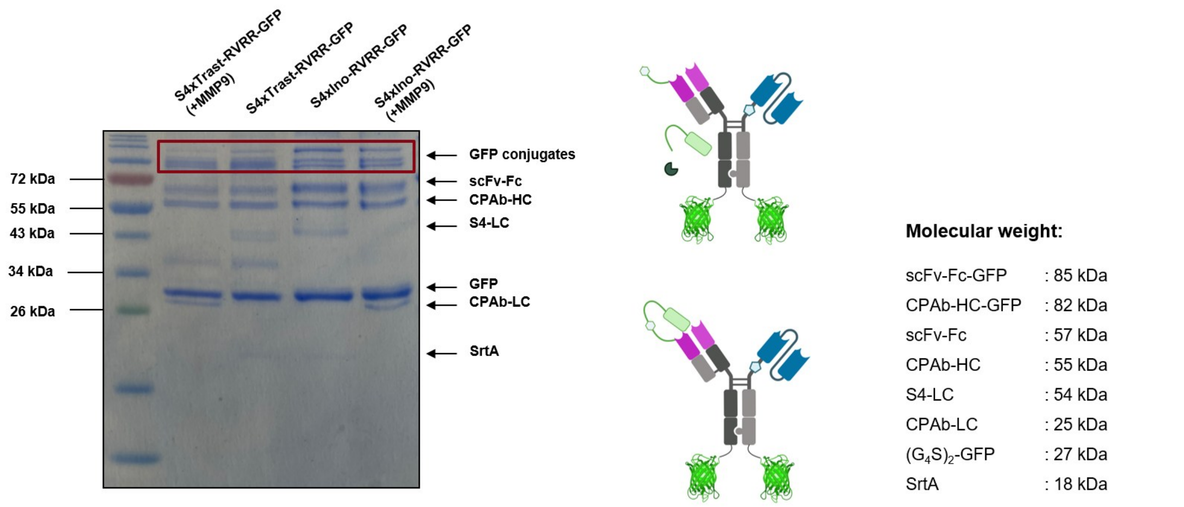

Figure S7. SDS-gel for the verification of the coupling reaction of eGFP with the bispecific constructs. S4xTrastuzumab-RVRR-eGFP and S4xInotuzumab-RVRR-eGFP (MMP-9 cleaved and uncleaved) were loaded on a reducing SDS-gel. The eGFP-coupled heavy chains are highlighted in red. Color Prestained Protein Standard, Broad Range (10–250 kDa) (NEB) was utilized as marker.

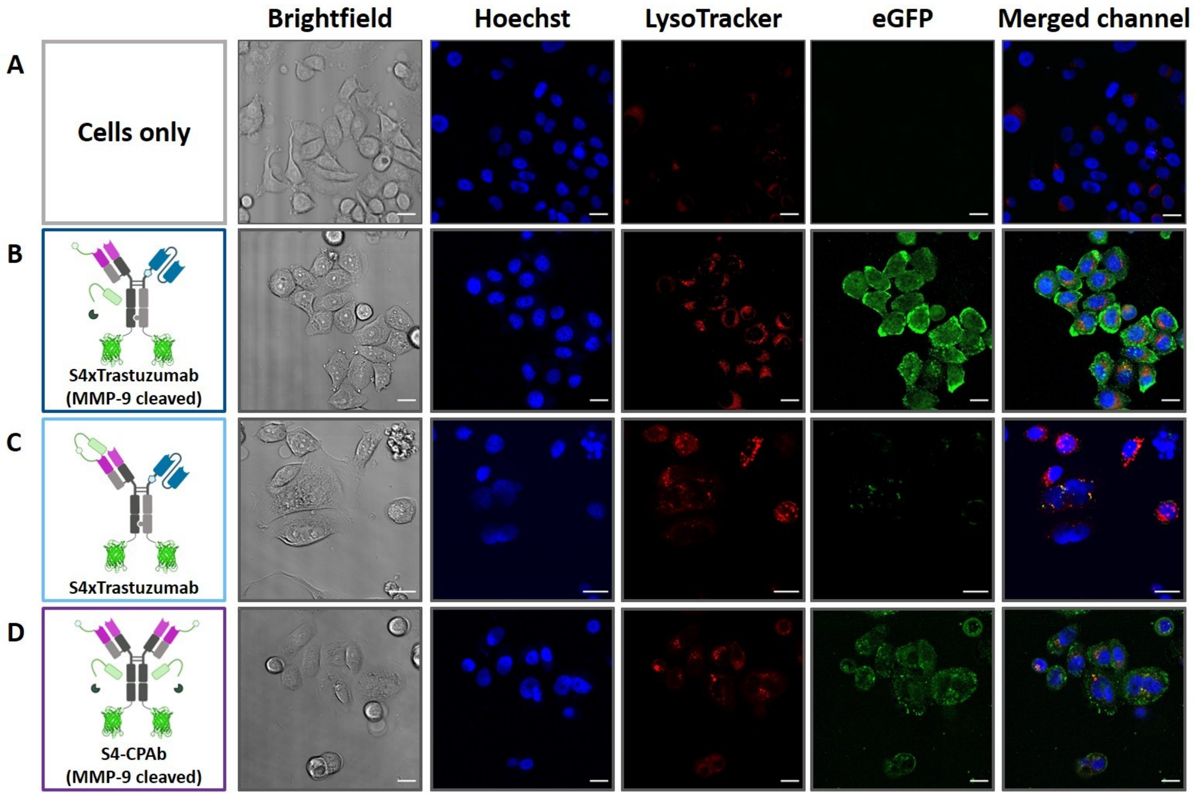

Figure S8. CLSM images of brightfield, Hoechst or GFP fluorescence channels of SKBR-3 cells treated with 1 µM antibody-GFP conjugates. (A) without antibody-eGFP conjugate, (B) S4xTrastuzumab-RVRR-eGFP (MMP-9 cleaved), (C) S4xTrastuzumab-RVRR-eGFP, and (D) bivalent S4 (MMP-9 cleaved) were incubated with SKBR-3 cells. The scale bar corresponds to 20 µm. Fluorescence images were generated with ImageJ 1.53c.

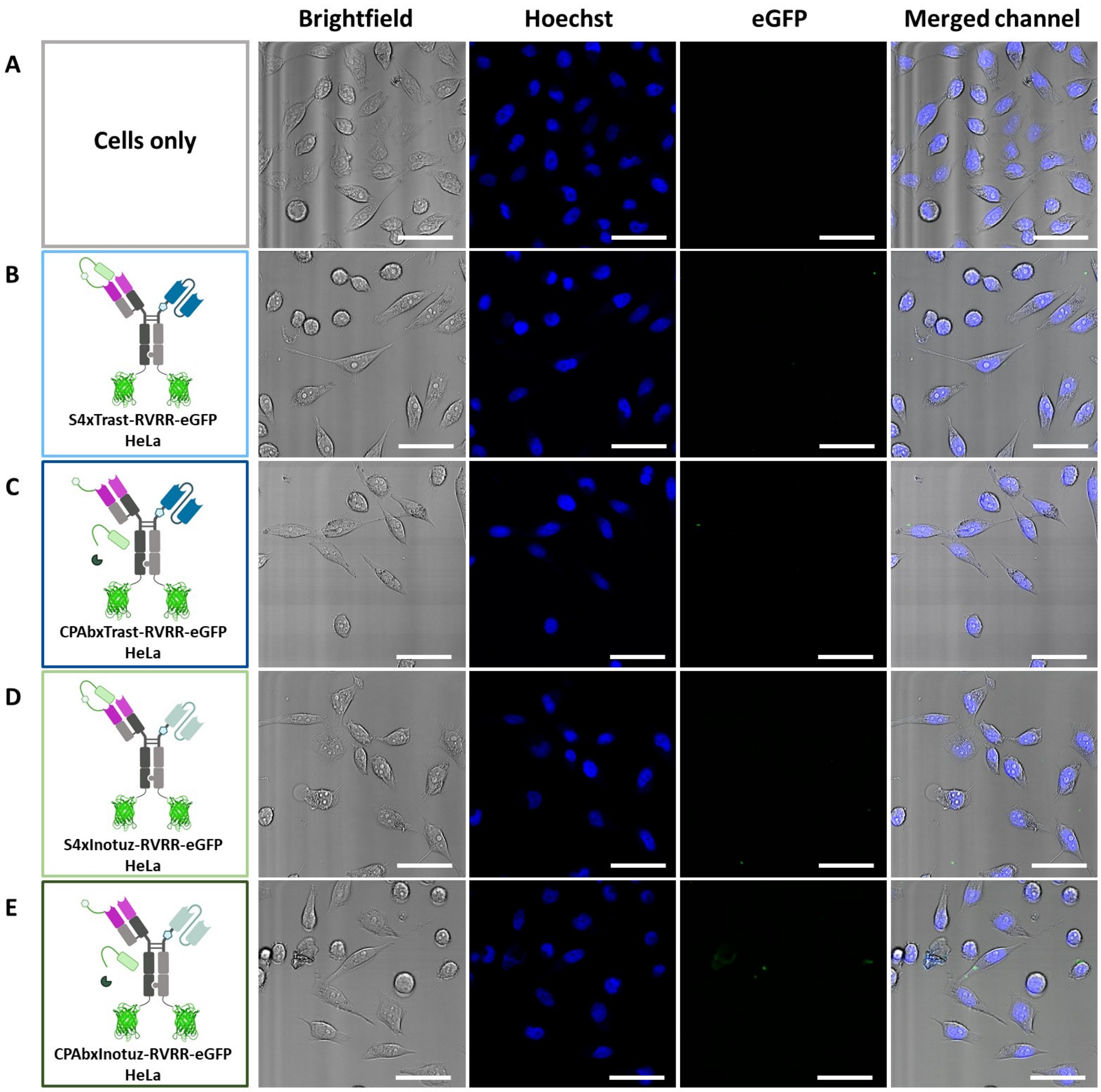

Figure S9: CLSM images of brightfield, Hoechst or GFP fluorescence channels of HeLa cells treated with 500 nM antibody-GFP conjugates. (A) without antibody-eGFP conjugate, (B) S4xTrastuzumab-RVRR-eGFP, (C) S4xTrastuzumab-RVRR-eGFP (MMP-9 cleaved), (D) S4xInotuzumab-RVRR-eGFP, and (E) S4xInotuzumab-RVRR-eGFP (MMP-9 cleaved) were incubated with HeLa. The scale bar corresponds to 50 µm. Fluorescence images were generated with ImageJ 1.53c.

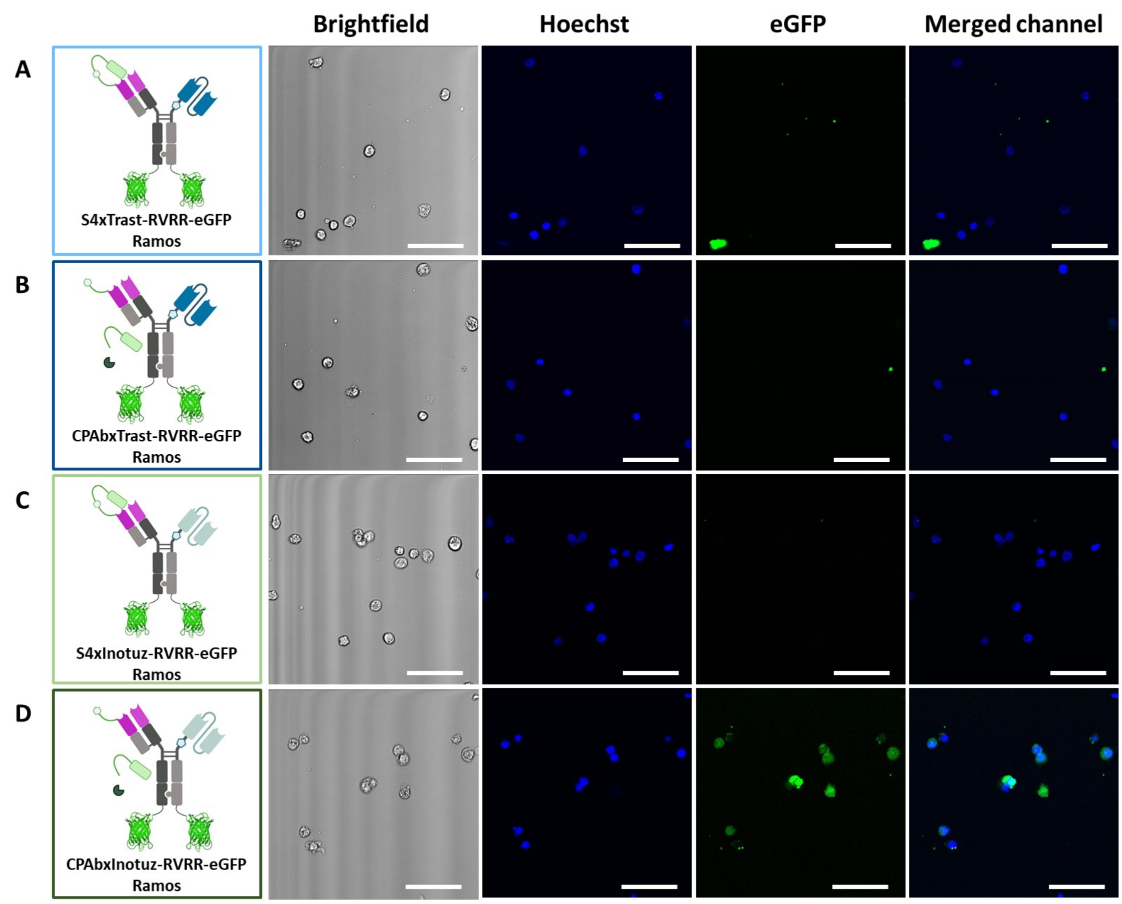

Figure S10. CLSM images of brightfield, Hoechst or GFP fluorescence channels of Ramos cells treated with 500 nM antibody-GFP conjugates. (A) S4xTrastuzumab-RVRR-eGFP, (B) S4xTrastuzumab-RVRR-eGFP (MMP-9 cleaved), (C) S4xInotuzumab-RVRR-eGFP, and (D) S4xInotuzumab-RVRR-eGFP (MMP-9 cleaved) were incubated with Ramos cells. The scale bar corresponds to 50 µm. Fluorescence images were generated with ImageJ 1.53c.

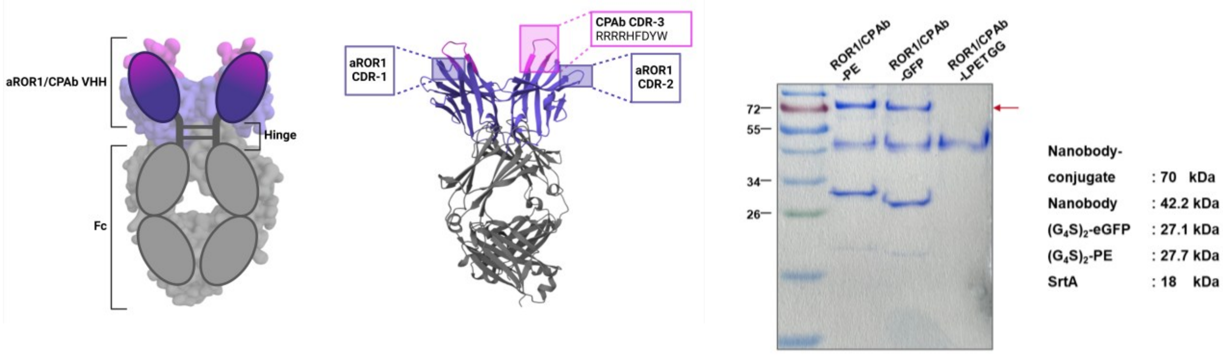

Figure S11. Schematic illustration of the VHH with exchanged CDR3 and SDS-gel of the construct before and after sortase A coupling reaction. Modeling of the VHH-Fc fusion was performed with alpha-fold. The native aROR1 CDR1-2 are highlighted in purple and the exchanged CDR3 in pink. The uncoupled VHH-Fc fusion, the PE_{cat}-respectively eGFP-coupled construct was loaded on a reducing SDS-gel. The coupled heavy chains are highlighted in red.

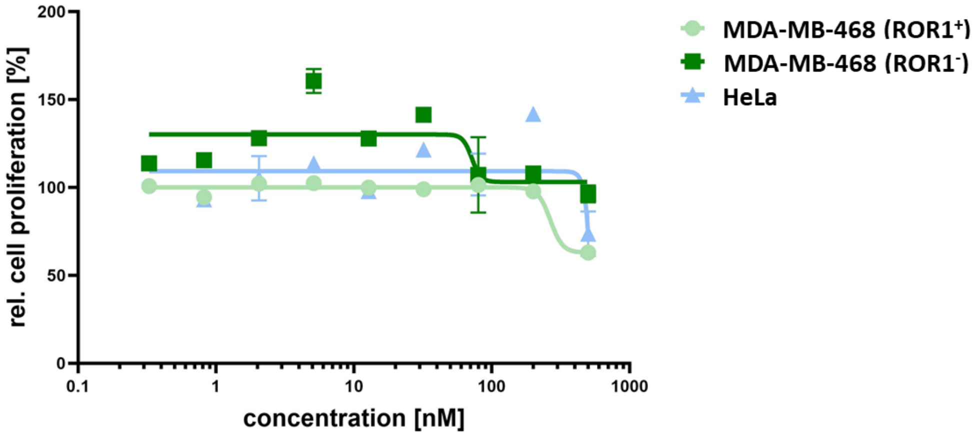

Figure S12. Determination of the cytosol-penetrating capabilities of the aROR1/CPAb VHH-Fc-PE via PE-mediated proliferation assay. The three different cell lines (MDA-MB-468 $ROR1^+$, MDA-MB-468 $ROR1^-$ and HeLa) were incubated with the PE-coupled construct in a dilution. The resulting data points are shown as mean and error bars that represent standard deviation derived from experimental duplicates and illustrated using GraphPad Prism 10.1.0 (316).

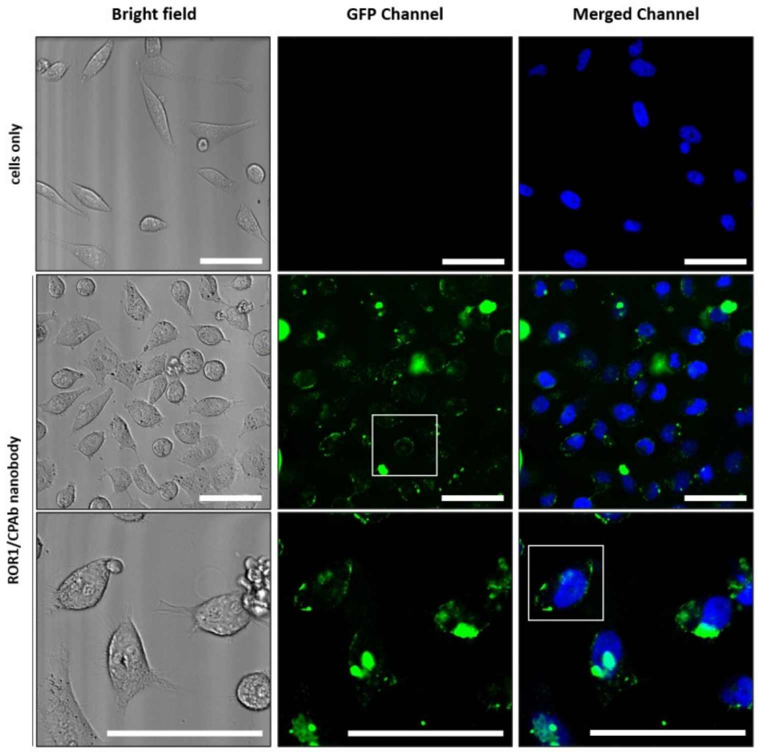

Figure S13. CLSM images of brightfield, Hoechst or GFP fluorescence channels of HeLa cells treated with 1 µM aROR1/CPAb-eGFP conjugate. Non-treated cells served as negative control. The scale bar corresponds to 50 µm. Fluorescence images were generated with ImageJ 1.53c.

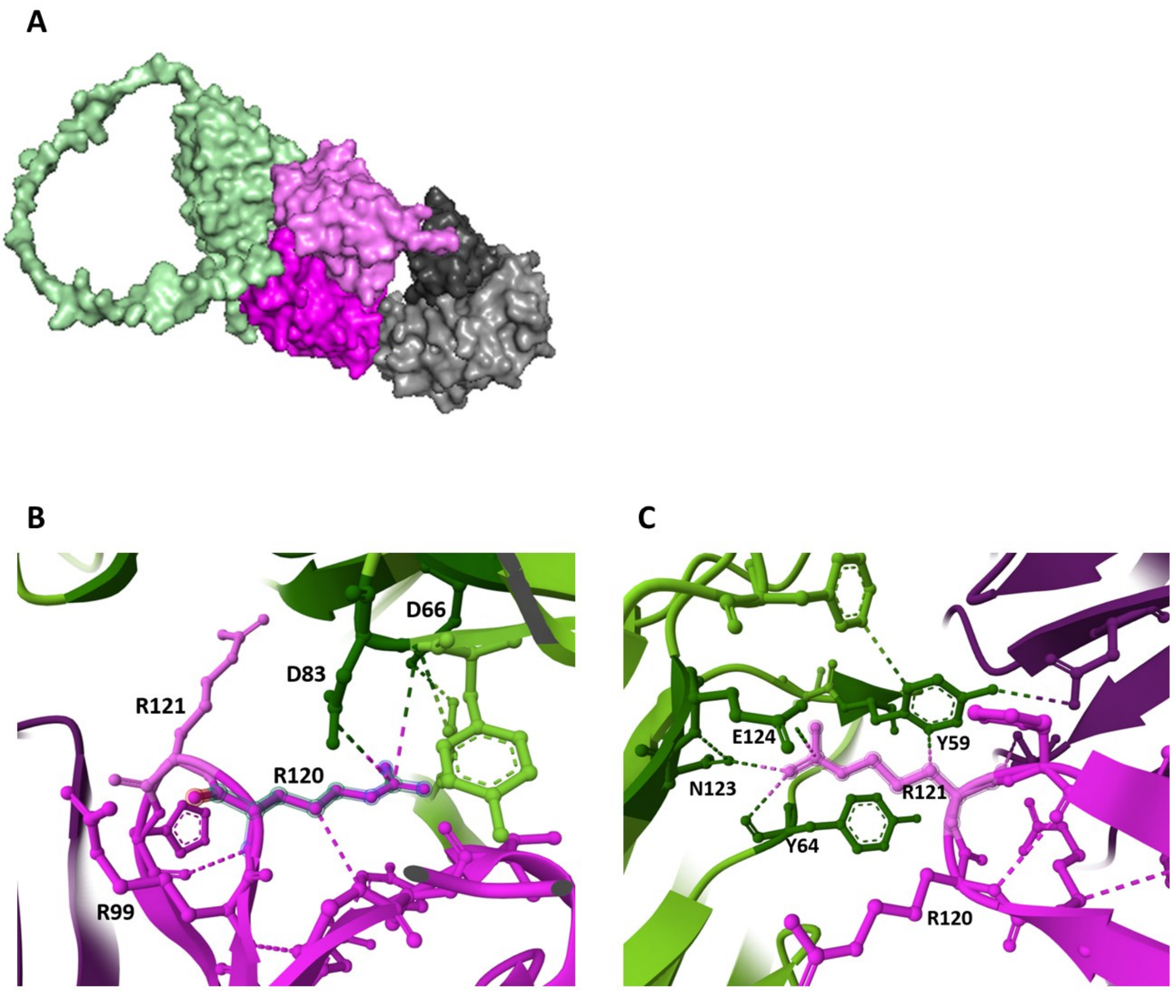

Figure S14. Modeled illustration of the masked CPAb Fab and the potential side chain interactions during the masking process. (A) Alpha-fold proposed modeling of the masking unit to the predominantly CPAb V_L. The masking moiety is shown in green, the CPAb variable domains in pink and the constant regions of the heavy and light chains in grey. (B) Ionic interactions between the Arg120 from the HSPG binding motive with Asp66 and Asp83 from the masking unit. (C) Proposed interactions of the mask with the Arg121 from CPAb V_L based on the alpha-fold model. Illustration via MolStar.
