# Supplementary material for: Tumor-specific cytosol-penetrating antibodies for antigen- and TME-dependent intracellular cargo delivery
Source: Mol Ther Oncol. 2025 Jan 2;33(1):200931. doi: 10.1016/j.omton.2024.200931 (PMC11786873; doi:10.1016/j.omton.2024.200931)
Supplement: Document S2. Article plus supplemental information [file mmc2.pdf]

Molecular Therapy
Oncology
Original Article

Tumor-specific cytosol-penetrating antibodies for antigen- and TME-dependent intracellular cargo delivery

Carolin Sophie Dombrowsky,[1] Felix Klaus Geyer,[1] Diana Zakharchuk,[1] and Harald Kolmar[1,2]

[1]Institute for Organic Chemistry and Biochemistry, Technical University of Darmstadt, Peter-Grünberg-Strasse 4, 64287 Darmstadt, Germany; [2]Centre for Synthetic Biology, Technical University of Darmstadt, 64287 Darmstadt, Germany

Although a considerable number of disease-related biomolecular interactions occur in the cytosol, therapeutic and diagnostic application of target-specific binding proteins is largely confined to surface-exposed or extracellular targets. Therefore, protein-cargo delivery approaches, including cell-penetrating peptides and cytosol-penetrating antibodies, are being explored to overcome this limitation. In this context, we have developed a modular approach for cytosolic penetration of tumor cells based on bispecific antibodies containing a masked cytosol-penetrating Fab on one arm and a tumor-targeting scFv linked via an endosomal cleavable linker on the other arm. The relevance of the antigen-specific binding, internalization, and cytosolic cargo delivery was demonstrated in several *in vitro* assays using different cell lines with anti-B7-H3 scFv, the well-characterized trastuzumab (HER2), and inotuzumab (CD22) as examples. In addition, presence of the masking moiety to prevent non-specific surface binding, as well as the activation of cytosol-penetrating capabilities in the tumor microenvironment upon release by tumor-specific proteases was confirmed using the catalytic domain of *Pseudomonas* exotoxin as model cargo for cytosol delivery. Tumor microenvironment-dependent as well as tumor-associated antigen-specific cytosol-penetrating antibodies of the type developed here have the potential to serve as a modular platform to deliver macromolecular cargoes for addressing intracellular targets in tumor cells.

INTRODUCTION

Although cancer is currently the second most common cause of mortality in the United States, behind only cardiovascular diseases, the current standard of care remains limited in its effectiveness.[1] Their ability to selectively target tumor cells is a highly desirable feature of many antibodies used in cancer therapy. However, the high molecular mass of conventional therapeutic antibodies, which is approximately 150 kDa, limits their ability to target tumor antigens to proteins or receptors that are presented on the cell surface.[2,3] Targeting of intracellular cancer-related proteins would represent a significant advancement in the field of disease diagnostics and treatment.[4] A physiological pathway by which antibodies can bypass the cellular outer membranes to reach the cellular interior is receptor-mediated endocytosis.[5] In this process, antibody molecules bind to specific receptors present on the external surface of cells when encountering tumor-associated antigens (TAA). This binding event subsequently leads to the internalization of the antibody-antigen complex into endosomal compartments.[6] This pathway predominantly results in either recycling to the cell surface or protease-dependent degradation of the receptor-antibody complex within lysosomes.[6]

Previously, antibodies with the capacity for endosomal escape have been identified and characterized. Cytosol-penetrating antibodies include the well-characterized TMab4 cytotransmab, as well as the recently discovered CPAb (cytosol-penetrating antibody).[7,8] Both antibodies are thought to follow a similar pathway to cytosolic localization.[8,9] In summary, they possess a heparan sulfate proteoglycan (HSPG) binding motif that includes a variety of positively charged amino acids (Lys, Arg) in the complementarity determining regions (CDRs), which induces receptor-mediated endocytosis upon binding.[10–12] The release of antibodies from the HSPG into the endosomes necessitates the involvement of endoglycosidase heparinase (HSPE).[9,13] HSPE is frequently overexpressed in tumor cells and functions to cleave heparan sulfate (HS) subsequent to its activation within the endosomes.[14,15] The precise mechanism of endosomal escape of this type of antibodies to prevent lysosomal degradation remains unclear. However, it is assumed that this process is initiated by a hydrophobic aromatic motif, such as WYW[9,16] or, as evidenced by our previously published CPAb, presumably HFDYW.[8] It is postulated that the pH decrease in the endosome induces local protonation, resulting in a conformational change and a differing relative orientation of the motif, thereby inducing interaction with the endosomal membrane and ultimately leading to the release of the antibody into the cytosol.[9] However, it is noteworthy that tumor cell-specific internalization does not occur with these approaches, as HSPG is present on the cell surface of the majority of cell types.[17]

Received 28 August 2024; accepted 30 December 2024;
https://doi.org/10.1016/j.omton.2024.200931.

Correspondence: Harald Kolmar, Institute for Organic Chemistry and Biochemistry, Technical University of Darmstadt, Peter-Grünberg-Strasse 4, 64287 Darmstadt, Germany.
E-mail: harald.kolmar@tu-darmstadt.de

Published by Elsevier Inc. on behalf of The American Society of Gene and Cell Therapy.
This is an open access article under the CC BY license (http://creativecommons.org/licenses/by/4.0/).

In a previous study, we described the development of a masked cytosol-penetrating antibody (S4-CPAb) aimed at establishing selective cell uptake in the tumor microenvironment (TME) (Figure 1A).[8] This antibody is designed to be conditionally activated preferentially in the TME through demasking by matrix metalloproteinase-9 (MMP-9) cleavage. We demonstrated that the masked antibody did not perform cytosol penetration on HeLa cells. However, following complete cleavage of the mask, the cytosol-penetrating properties of the antibody were fully restored.

To accumulate an antibody on tumor cells and further enhance tumor selectivity, we reasoned that it might be desirable to generate an internalizing antibody carrying an additional tumor-targeting module. To date, few approaches have been established to enable tumor cell targeting in combination with cytosol penetration and cargo delivery. The implementation of these approaches requires several critical features, such as (1) implementation of a tumor cell-selective binding module, (2) conditional accessibility of the heparan sulfate proteoglycan to prevent non-specific HSPG-dependent internalization, and (3) release of the antibody or the cargo from any membrane-bound target molecules in the endosome to allow endosomal escape and release into the cytosol (Figure 1C).

In this study, we developed a modular approach to generate TAA-specific, cytosol-penetrating antibodies based on the masked S4-CPAb, with the objective of combining high binding affinity, efficient internalization, and endosomal escape. We envisioned a bispecific antibody with one arm responsible for tumor cell binding, while the other arm mediates cytosolic localization and cargo delivery (Figure 1B). To this end, we generated various bispecific antibodies, wherein one arm contained the masked S4-CPAb Fab and the other arm a TAA-binding single-chain variable fragment (scFv) targeting HER2, CD22, and B7-H3, respectively. Endosomal proteolytic cleavage by furin enabled release from the membrane-bond receptor and eventually translocation of the truncated antibody together with its cargo into the cytosol. Two orthogonal assays confirmed both, the tumor cell-specific cell permeability of the constructs and their ability to deliver protein cargo. In addition, the relevance of the receptor-mediated internalization rate induced by antigen binding and the requirement for proteolytic release of the endosomal escape module from the target receptor was investigated.

We demonstrate a modular approach for the generation of multifunctional antibodies with tumor-associated antigen-specific internalization via antibody fragments, combined with conditionally activatable (TME-depended) cytosol penetration, resulting in cytosolic cargo localization dependent on both tumor cell-specific binding and TME cleavage.

RESULTS

Design of bispecific tumor cell-targeting cytosol-penetrating antibodies

Building upon the previously published bivalent CPAb, which contains the masking unit S4 (Figure 1A[8]) we have designed bispecific antibodies with an additional tumor-targeting domain. This approach aims to achieve tumor cell-specific cytosol penetration and, simultaneously, an increase in affinity. Our approach involves genetically fusing the S4-CPAb Fab to a specially designed "hole"-Fc, and the "knob"-Fc to different TAA-targeting scFvs, thereby verifying the modular approach (Figure 2). The knobs-into-holes technique allows for easy asymmetric self-assembly of half antibodies upon protein expression.[18] The three tumor-targeting proteins that were tested were single-chain variable fragment (scFv) variants of the full-length antibodies of an in-house screened anti-B7-H3,[19] inotuzumab, a clinically approved CD22 binder,[20,21] and the well-characterized HER2-binding trastuzumab.[22,23] Previous studies have demonstrated the rapid and efficient internalization of the parental inotuzumab[24,25] and trastuzumab.[26,27] Based on this evidence, our assumption was that despite monovalent binding of the scFvs, a high target affinity and rapid internalization could lead to an increased intracellular concentration of the unmasked antibodies. Furthermore, to confirm the pivotal role of endosomal release of the monovalent cytosol-penetrating unit from the receptor complex, we designed different linkers containing a furin cleavage site (endosomal cleavage[28]), a GS linker (non-cleavable), and a legumain cleavable linker (lysosomal cleavage[29,30]). The different linker sequences, along with the proposed mode of action, are illustrated in Figures 2 and 1C, respectively.

On-cell-binding and tumor-target-associated internalization

To investigate the tumor-associated antigen-specific affinity of the bispecific antibody using Trastuzumab scFv (S4xTrastuzumab) on HER2-positive SKBR-3[31] and HER2 low-expressing HeLa cells,[32] we conducted on-cell-binding assays using flow cytometry (Figure 3A). The masked construct demonstrated concentration-dependent binding exclusively on SKBR-3 cells, with an apparent K_D of 38 nM. Conversely, binding on HeLa cells was not observed at concentrations up to 2 μM. To ascertain the impact of the HSPG-binding domain of the CPAb Fab on cell-specific binding, S4xTrastuzumab was unmasked via MMP-9 cleavage and tested on HeLa and SKBR-3 cells. In comparison to the masked constructs, a slight decrease in the maximum mean fluorescence intensity (MFI) and improved apparent K_D (17 nM) was observed with SKBR-3 cells upon mask release. Conversely, an unmasked isotype control (CPAbxαB7-H3) only showed slight binding at the highest concentration on SKBR-3 cells. Together with additional binding assays to compare the masked and unmasked constructs, including S4xαB7-H3 and S4xTrastuzumab on HeLa (Figure S1B) and S4xInotuzumab on Ramos cells (Figure S1A), this further corroborates the notion that tumor cell binding is primarily dependent on the scFv module and that the masking moiety is cruel to prevent non-specific tumor-associated target binding. Subsequent binding assays were conducted with the previously published bivalent CPAb and the masked S4-CPAb on the HSPG-positive HeLa and SKBR-3 cells and the HSPG-negative Ramos cells, which demonstrated the tumor cell non-specific binding of the bivalent constructs in comparison with the bispecifics (Figure S1C).

Similar binding assays were conducted with the masked constructs S4xTrastuzumab, S4xInotuzumab, and S4xαB7-H3 and tested on three different cell lines: HeLa (B7-H3[+33,34]), SKBR-3 (HER2[+31]),

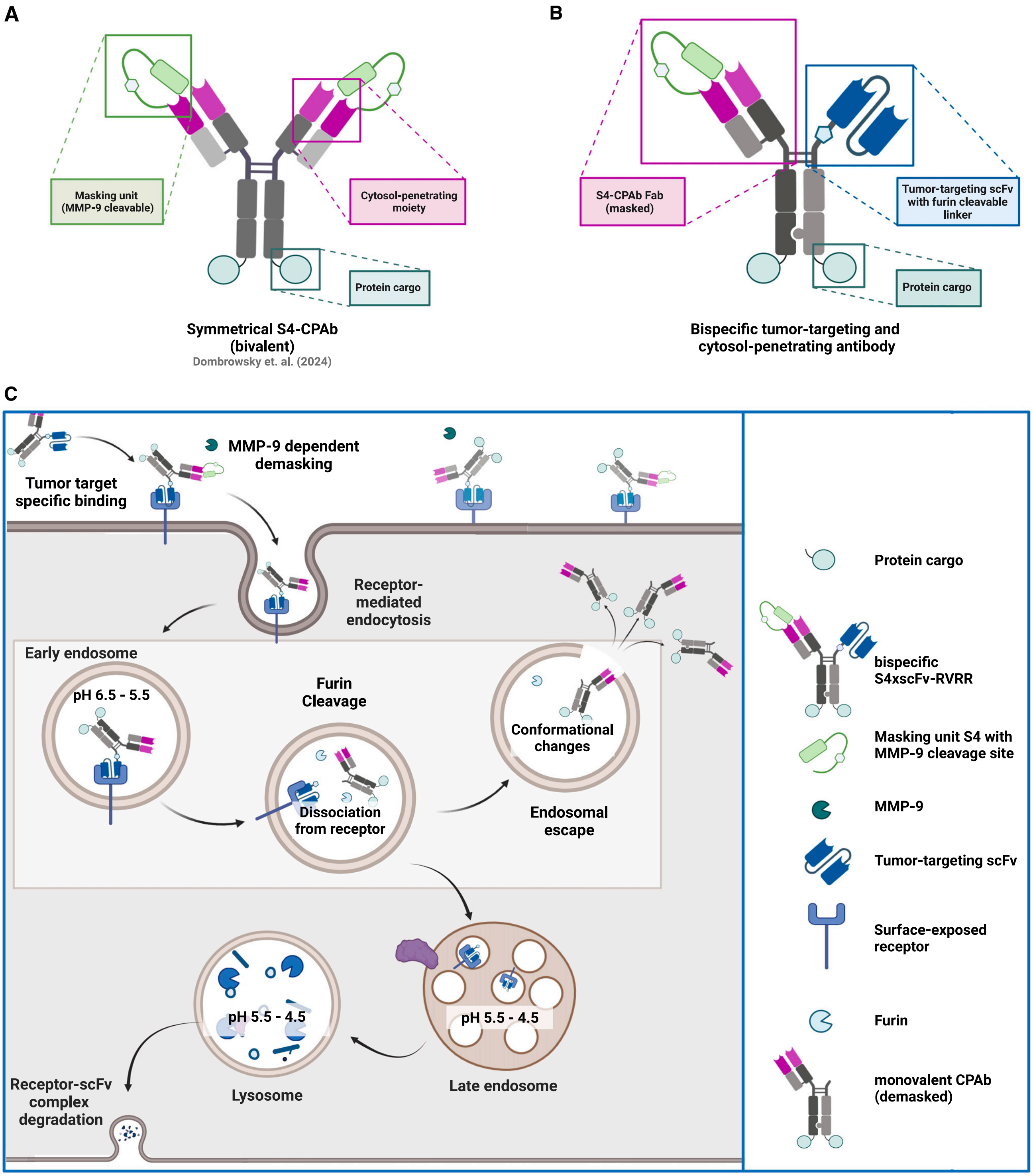

Figure 1. Design and mode of action of tumor cell targeting cytosol-penetrating antibodies

(A) Schematic illustration of the previously published, conditional activatable cytosol-penetrating antibody S4-CPAb.[8] (B) Illustration of the design of bispecific antibody constructs. The masked CPAb-Fab fragment is C-terminally fused to the hinge of hole-Fc, while the tumor-targeting scFv is fused to the hinge region of knob-Fcs via a cleavable linker. The C-terminal LPETGG tag allows for sortase A-mediated coupling with toxins or fluorophores. (C) Illustration of the pathway following MMP-9 cleavage and antigen-specific internalization. Furin cleavage in the early endosome leads to endosomal release of the one-armed CPAb construct, allowing endosomal escape. The scFv-receptor complex is degraded via the lysosomal pathway. The figure was created using BioRender.com.

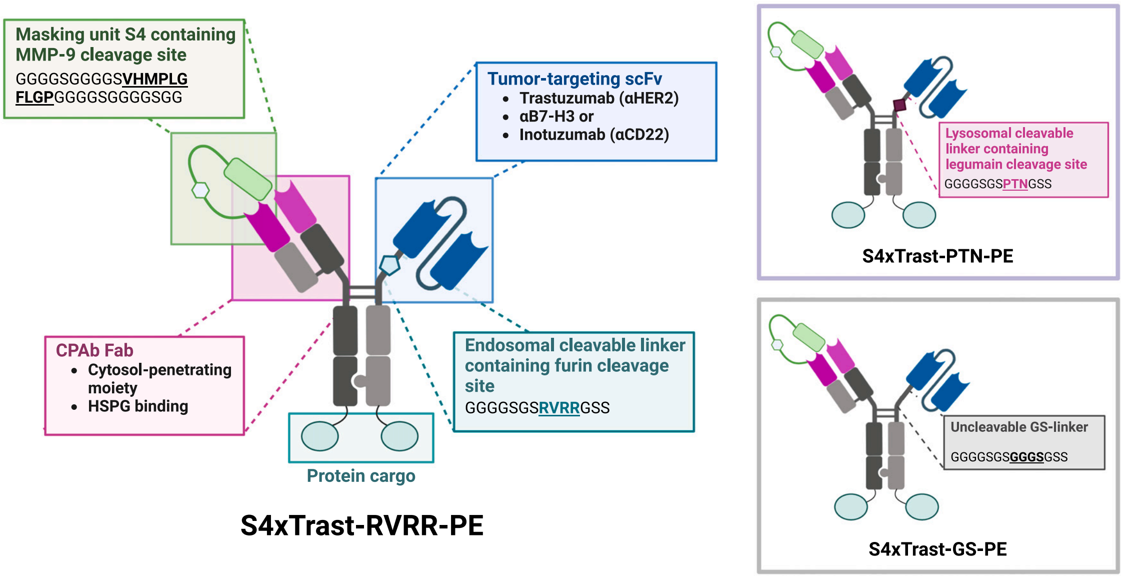

Figure 2. Design of bispecific antibody constructs, exemplified by S4xTrastuzumab

The masked CPAb-Fab fragment is C-terminally fused to the hinge of hole-Fc, while the tumor-targeting scFv is fused to the hinge region of knob-Fcs via a cleavable linker. The C-terminal LPETGG tag allows for sortase A-mediated coupling with toxins or fluorophores. The figure was created using BioRender.com.

and Ramos (CD22^{+}[35]). The results of the binding assay of the bispecific constructs on SKBR-3 cells demonstrated, as anticipated, that the B cell receptor-targeting S4xInotuzumab exhibited no binding on the breast cancer cell line, while S4xαB7-H3 led only to a slight increase in MFI at the highest concentration (Figure 3B). This may be attributed to the general wide distribution of the target on different tumor cell lines. No on-cell-binding was observed for S4xInotuzumab on CD22-negative and HER2 low-expressing HeLa cells (Figure 3C). Furthermore, nonsignificant binding was detected for S4xTrastuzumab on HeLa cells, which are known to be HER2 low and B7-H3 high, while an effective concentration of 25 nM could be determined for S4xαB7-H3. The B-lymphocyte cell line Ramos exhibited binding only to the CD22-targeting S4xInotuzumab, with a K_D of 3 nM (Figure 3D). In conclusion, the results demonstrate that the masked constructs exhibit antigen-dependent and specific binding exclusively to the corresponding receptor-positive cell line.

Subsequently, we aimed to determine the TAA-dependent internalization capabilities of the bispecific constructs in comparison with the bivalent initial constructs trastuzumab and αB7-H3. The bivalent CPAb served as the positive control, while the mutated variant, which lacks the HSPG-binding motif CPAb (118S-121S), served as the negative control. To investigate the internalization capabilities, the antibodies were coupled with a pH-sensitive fluorescent dye, which emits a minimal fluorescence signal at neutral pH values and an increased signal at acidic pH values (endosomes, lysosomes).[36] The antibodies were incubated in a dilution series ranging from 0.2 nM to 500 nM for 24 h with the respective cell line. Efficient internalization of trastuzumab (half maximal effective concentration [EC_{50}]: 1.9 nM) and native CPAb were confirmed on the HER2- and HSPG-positive SKBR-3 cells (Figure 4A). As anticipated, the non-binding negative control did not exhibit a notable increase in MFI. However, for the bispecific S4xTrastuzumab construct, a concentration-dependent increase in internalization was confirmed despite monovalency, resulting in an effective concentration of 28 nM, which is comparable to the K_D of 38 nM. A comparison of the parent trastuzumab and S4xTrastuzumab indicates a 15-fold increase in the effective concentration and a reduction in the maximum MFI by 30%. In the comparative experiment on HeLa cells, neither the monovalent S4xαB7-H3 nor the native αB7-H3 antibody demonstrated a notable increase in MFI, and consequently internalization rate, in comparison to the negative control CPAb (118S-121S) (Figure 4B). Internalization could only be detected in HeLa cells for the native CPAb, which served as a positive control, manifesting in a 20-fold and 10-fold increase in the area under the curve (AUC) compared with CPAb (118S-121S) and αB7-H3, respectively (Figure 4D). Finally, the TAA-associated internalization rates of CPAb, CPAb (118S-121S), and S4xInotuzumab were compared in the HSPG-negative Ramos cells (Figure 4C).[37] No internalization was observed for CPAb and the mutated CPAb variant. However, a notable increase in MFI was evident for S4xInotuzumab, resulting in an EC_{50} value of 184 nM. In a final cross-cell comparison of the bispecific constructs, S4xTrastuzumab and S4xInotuzumab exhibited comparable, normalized AUC values despite markedly disparate EC_{50} values (Figure 4D). It is noteworthy that comparative internalization assays of the bispecific constructs after cleavage of the masking unit for the highly internalizing constructs S4xTrastuzumab (Figure S2A) and S4xInotuzumab (Figure S2C) demonstrated a reduction of the maximum internalization and AUC values by approximately 50%, which could be attributed to the subsequent CPAb-dependent endosomal escape of the unmasked

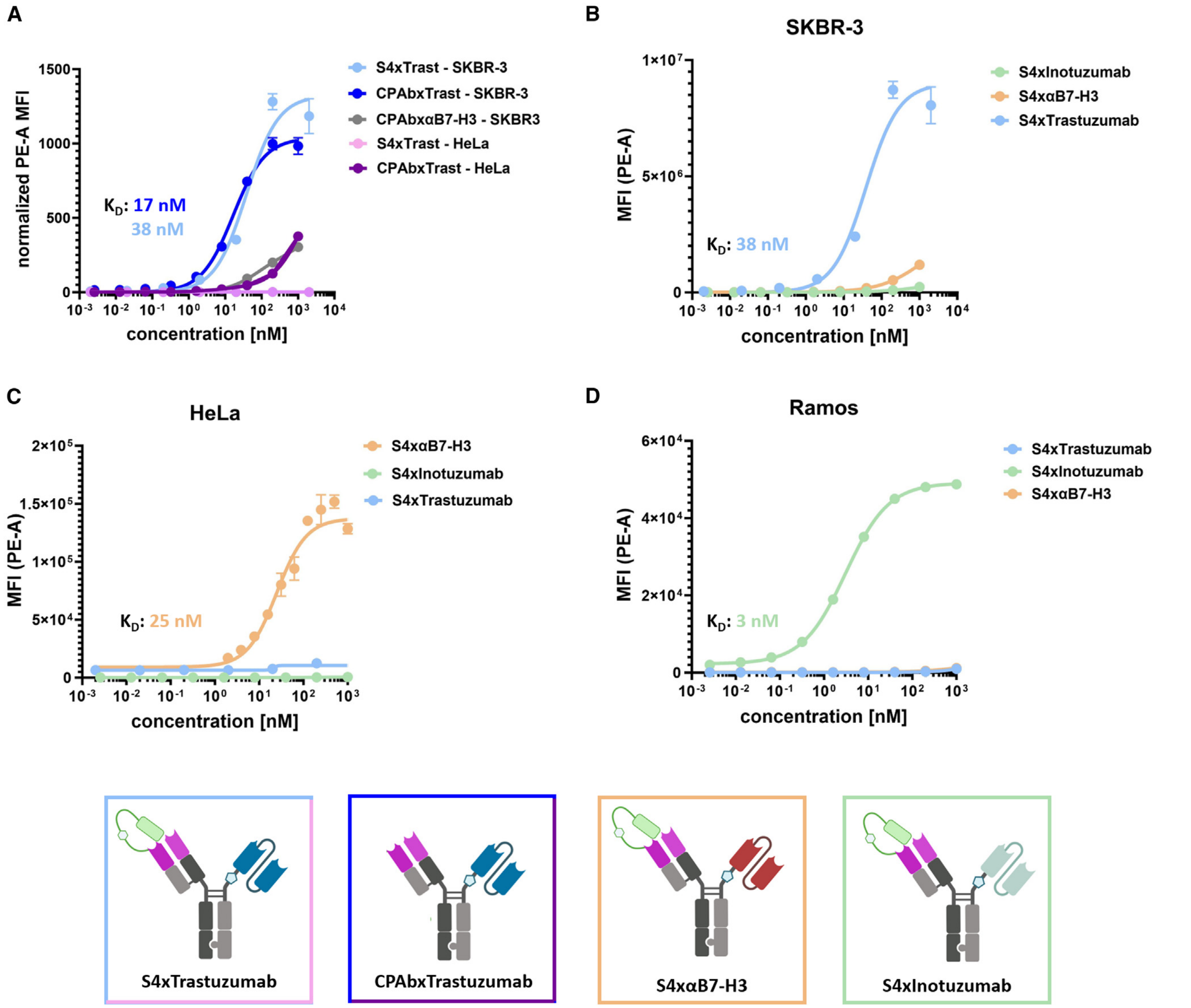

Figure 3. On-cell binding assay of the bispecific using SKBR-3, HeLa and Ramos cells

The cells were treated with concentrations of the compound ranging from 0.002 nM to 2000 nM. (A) A comparative analysis of the impact of non-specific HSPG binding on binding to SKBR-3 and HeLa, exemplified by S4xTrastuzumab and S4xαB7-H3 as isotype control. Evaluation of on-cell binding of the bispecific constructs S4xTrastuzumab, S4xαB7-H3, and S4xInotuzumab on (B) SKBR-3, (C) HeLa, and (D) Ramos cells, respectively. The K_D values were calculated from variable slope four-parameter fitting using GraphPad Prism 10.1.0 (316) and presented in the corresponding graph. Results are shown as mean, and error bars represent standard deviation derived from experimental duplicates.

constructs. In the case of the poorly internalizing B7-H3-targeting bsAb, the cleavage of the mask did not result in any discernible effect (Figure S2B), which lends support to the hypothesis that both cell binding and internalization are predominantly TAA-mediated.

Investigation of *in vitro* cytosol penetration capabilities

Influence of the linker cleavability on the cytosol-penetrating capabilities

To investigate the impact of the endosomal release of the receptor-binding module on endosomal escape of the CPAb carrying a cargo protein (Figure 1C), PE-mediated cytosol penetration assays were conducted with diverse S4xTrastuzumab constructs. When entering the cell cytosol, the catalytic domain of *Pseudomonas* exotoxin (PE_{cat}) blocks protein synthesis by ADP ribosylation of elongation factor 2. PE_{cat} is a truncated version of *Pseudomonas* exotoxin A, which has been genetically engineered to lack the receptor-binding domain, translocation domain, and other signal sequences such as KDEL. Consequently, only the active transport PE_{cat} by a carrier protein directly into the cytosol results in the regeneration of the toxin's cytotoxic capabilities. Hence, concentration-dependent cell killing can be

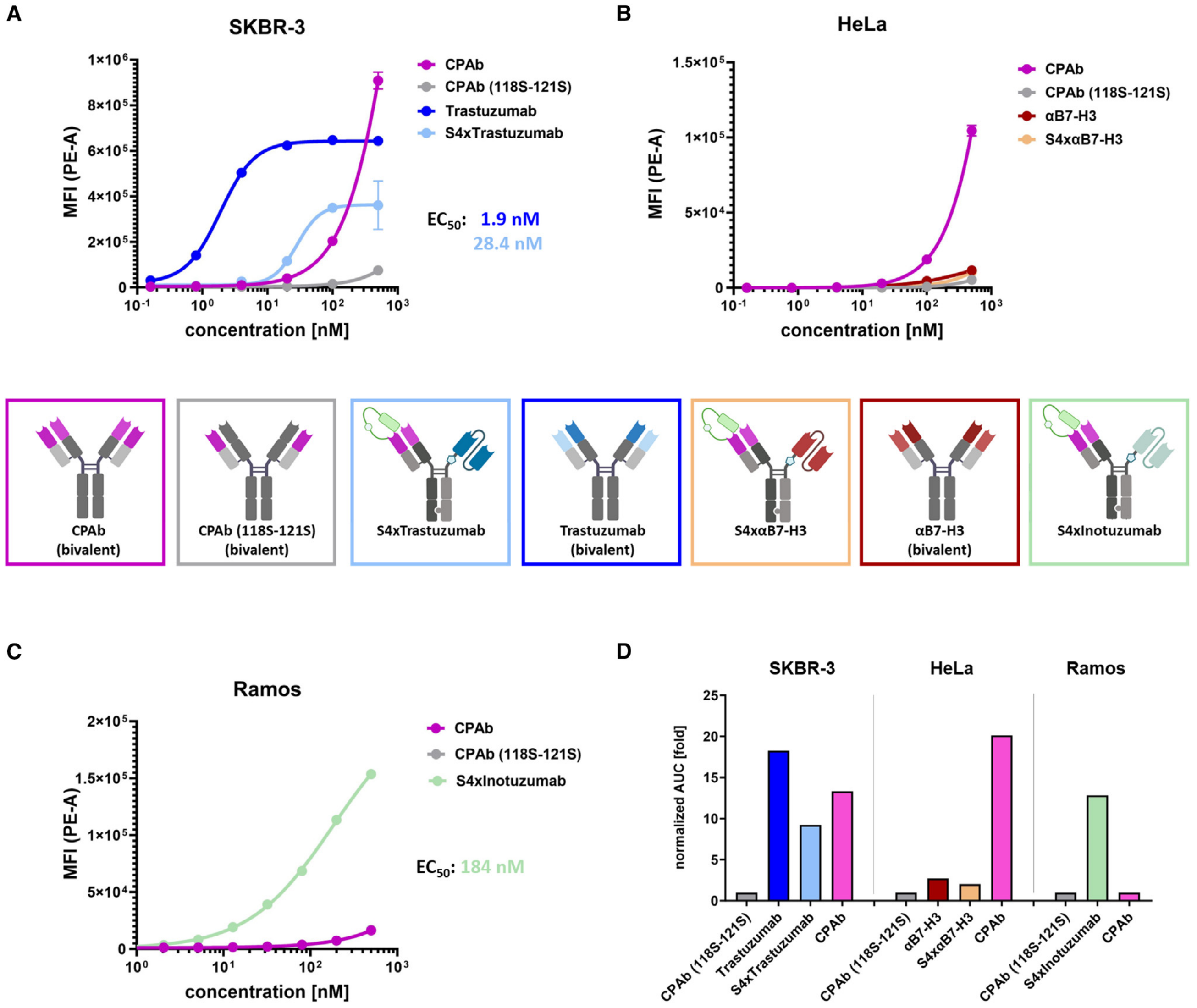

Figure 4. Determination of antigen-dependent internalization of the bispecific constructs in comparison to the corresponding native bivalent antibodies

The respective cell lines (A) SKBR-3, (B) HeLa, and (C) Ramos were incubated with the antibody-dye conjugates for 24 h. (D) The visualization of the area under the curve of the constructs, normalized to CPAb (118S-121S), allows for a comparison of the amount of internalizing antibodies. The EC_{50} values were determined from a four-parameter variable slope fit using GraphPad Prism 10.1.0 (316). Results are shown as mean, and error bars represent standard deviation derived from experimental duplicates.

used as an indicative of cytosolic localization of the PE_{cat} cargo.[8] Three different linkers were used to fuse the HER2 binding scFv module to the masked, one-armed CPAb antibody. These consisted of a non-cleavable GS linker, an endosomal cleavable (furin cleavable; RVRR[38]), or a lysosomal-cleavable (legumain cleavable; PTN[39]) linker, respectively (Figure 5). To these three bispecifics, PE_{cat} was enzymatically conjugated as previously published in Dombrowsky et al.,[8] via a sortase A coupling reaction. The cytosol-penetrating capabilities of the aforementioned S4xTrastuzumab-PE constructs were evaluated in a concentration range from 0.15 nM to 100 nM on SKBR-3 cells, prior to and following MMP-9 cleavage (Figure 5). The constructs with a non-cleavable linker (S4xTrast-GS-PE and S4xTrast-GS-PE [+MMP-9]) demonstrated no notable impact on cell viability, irrespective of whether they were masked or unmasked. The construct with a predominantly lysosomal-cleavable linker (S4xTrast-PTN-PE) demonstrated comparable cytotoxic effects before and after MMP-9 cleavage, resulting in a maximum killing efficiency of approximately 30%, and a single-digit nanomolar EC_{50}. When the data with the furin-cleavable linker are considered, a significant difference between the masked and unmasked variants is evident. Although the EC_{50} values are comparable (4.7 nM and 4.0 nM, respectively), a significant reduction in cell viability to 14% can be observed with the unmasked S4xTrast-RVRR-PE construct. This is additionally elucidated by a comparison of the normalized

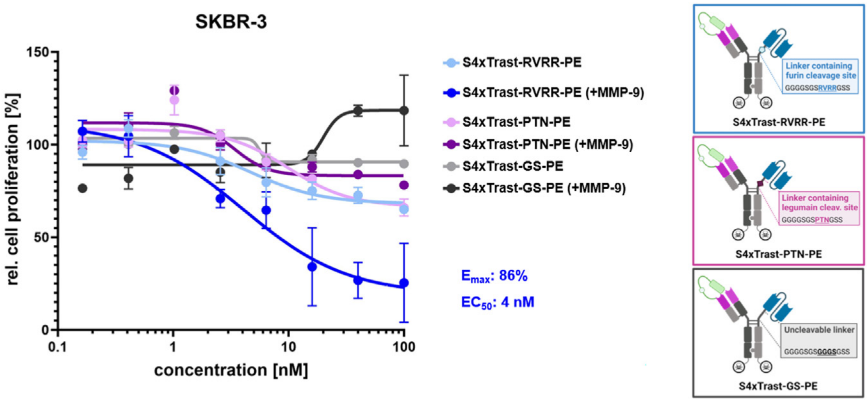

Figure 5. Determination of linker-dependent cytosolic penetration of S4xTrastuzumab constructs in SKBR-3 cells

Proliferation assay of S4xTrastuzumab-RVRR-PE_{cat} (MMP-9 cleaved and untreated) compared with masked and unmasked S4xTrastuzumab-GS-PE_{cat} (non-cleavable) and S4xTrastuzumab-PTN-PE_{cat} (Legumain cleavable) in SKBR-3 cells at different concentrations (0.15–100 nM). EC_{50} values were determined from a four-parameter variable slope fit using GraphPad Prism 10.1.0 (316). Results are shown as mean, and error bars represent standard deviation derived from experimental duplicates

AUC (Figure S3A), which revealed comparable AUC values for all constructs, with the exception of the unmasked S4xTrastuzumab-RVRR-PE, whose resulting area is 60% lower than of the other constructs. This may prevent endosomal membrane adherence via receptor binding and eventually lysosomal degradation of the entire construct. One potential explanation for the comparable EC_{50} values is the general proliferation-inhibiting effect of trastuzumab on SKBR-3 cells.[40] Given that only the construct with a furin cleavage site led to a reduction in cell viability, subsequent experiments focused on constructs with RVRR-including furin cleavage site.

Tumor cell-specific cytosol penetration

To investigate antigen-specific cytosol penetration, S4xTrastuzumab, S4xαB7-H3, and S4xInotuzumab were coupled C-terminally with PE_{cat}, the catalytic domain of *Pseudomonas* exotoxin PE, via a sortase A reaction. A coupling rate of approximately 60% was determined, and no preference for one of the heavy chains was detected (Figure S4), indicating that a heterogeneous distribution of toxins between 0 and 2 toxins per antibody in the population can be assumed.[8]

In contrast to the previously described S4xTrastuzumab-RVRR-PE and the bivalent unmasked S4-CPAb, neither the αB7-H3 nor the inotuzumab variants demonstrated a proliferation-inhibiting effect in SKBR-3 cells at the concentration range tested (Figure 6A). As demonstrated by analysis of the AUC (Figure S3B), the demasked S4xTrastuzumab constructs exhibit a significantly enhanced level of efficacy when compared with the CD22-or B7-H3-binding constructs and even when benchmarked against the previously published S4-CPAb. The results of the cytosol penetration assay in HeLa cells (Figure 6B) demonstrate that the S4xInotuzumab constructs do not inhibit proliferation, and that the trastuzumab construct exerts only a slight effect at the highest concentration (37% maximum killing efficiency). The masked S4xαB7-H3 variant revealed a comparable cytotoxic effect to that of the masked S4xTrastuzumab constructs (E_{max}: 45%), whereas the unmasked variant, following MMP-9 cleavage, resulted in complete cell killing, albeit with a high EC_{50} value of 326 nM in comparison to the previously published demasked S4-CPAb (EC_{50}: 55 nM). Furthermore, the analysis of the AUC demonstrates a specific effect associated with the S4xαB7-H3-RVRR, although it is less pronounced than the effect of the unmasked S4-CPAb (Figure S3C).

To further substantiate the cytosol penetration capabilities of the unmasked S4xαB7-H3 variant, a split-luciferase-based NanoBiT assay was conducted,[8] employing a HeLa cell line that inducibly expressed intracellular LgBiT luciferase protein that can be functionally complemented by HiBiT peptide fused to the cell-penetrating antibodies (200 nM) (Figure S5). Native bivalent CPAb-HiBiT and the S4xTrastuzumab-HiBiT were utilized as positive and negative controls, respectively. The utilization of a cell-permeable substrate enables the real-time detection of cytosolic localization subsequent to intracellular complementation of LgBiT protein and the HiBiT peptide, obviating the necessity for prior cell lysis.[41] Expectedly, unmasked S4xTrastuzumab displayed significantly lower luminescence signal in $HER2_{low}$/B7-$H3_{high}$ HeLa cells compared with unmasked S4xαB7-H3. In comparison to the bivalent, native CPAb, S4xαB7-H3 resulted in a slightly diminished yet analogous luminescence signal.

The masked constructs demonstrated no discernible inhibitory effect on cell proliferation within a concentration range of 0.15 nM–100 nM in Ramos cells. Nevertheless, cell killing was observed for all unmasked cell penetration mediating constructs, although the EC_{50} values differed considerably (Figures 6C and 6D). With an EC_{50} value of 1.2 nM and a maximum killing efficiency of nearly 90%, the CD22-targeting S4xInotuzumab is markedly more efficacious than the two isotype controls, which also showed cell killing at higher concentrations. The comparison with the previously published S4-CPAb demonstrated results analogous to those observed with the non-binding S4xαB7-H3, as Ramos cells are deficient in HSPG. As additionally demonstrated by the analysis of the AUC (Figure S3D), the unmasked S4xInotuzumab construct exhibited cell specificity in comparison to the isotype controls and S4-CPAb. Notably, a mixture of MMP-9 and PE_{cat} enzyme alone, which are present in the antibody-conjugate preparation to some extent, resulted in cytotoxicity at higher concentrations (Figure S6), which most likely accounts for the observed Ramos cell killing by isotype controls.

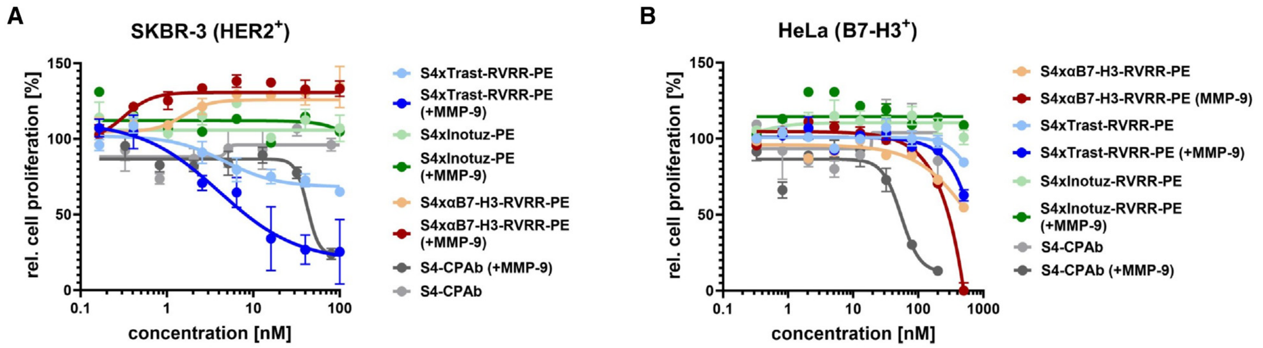

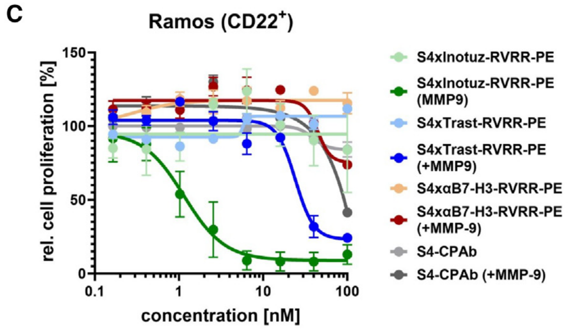

D

	SKBR-3		HeLa		Ramos	
	EC_{50}	E_{max}	EC_{50}	E_{max}	EC_{50}	E_{max}
S4xTrast	4.7	33 %	n. d.	15 %	n. d.	-
S4xTrast (+MMP-9)	**4 nM**	**86 %**	n. d.	37 %	24 nM	80 %
S4xαB7-H3	n. d.	-	n. d.	45 %	n. d.	-
S4xαB7-H3 (+MMP-9)	n. d.	-	**326 nM**	**100 %**	45 nM	36 %
S4xInotuz	n. d.	-	n. d.	-	n. d.	-
S4xInotuz (+MMP-9)	n. d.	-	n. d.	-	**1.2 nM**	**87 %**
S4-CPAb	n. d.	-	n. d.	-	**n. d.**	-
S4-CPAb (+MMP-9)	**42 nM**	**72%**	**55 nM**	**75 %**	**n. d.**	**60 %**

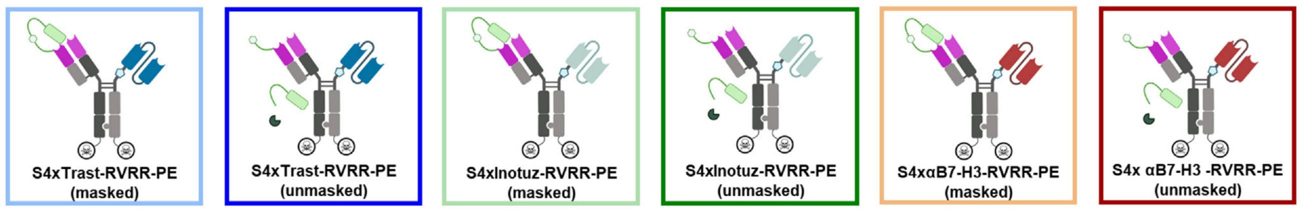

Figure 6. Cell-specific cytosol penetration capabilities of the bivalent S4-CPAb-PE and bispecific antibody-PE conjugates (MMP-9 cleaved and uncleaved) determined by PE-mediated proliferation assay

S4-CPAb, S4xTrastuzumab-RVRR-PE, S4xInotuzumab-RVRR-PE, and S4xαB7-H3-RVRR-PE were tested in (A) SKBR-3, (B) HeLa, and (C) Ramos cells. EC_{50} values were determined from a four-parameter fit with variable slope using GraphPad Prism 10.1.0 (316) and are tabulated in (D) together with the maximum killing effect. Results are shown as mean, and error bars represent standard deviation derived from experimental duplicates.

In conclusion, the PE-mediated proliferation assays for the detection of cytosol penetration demonstrated an overall tumor cell and target-specific cytosol penetration. The use of the tumor-target-binding scFvs trastuzumab and inotuzumab appears to enhance tumor cell specificity and to result in an increased intracellular antibody concentration in comparison to the previously published bivalent S4-CPAb. Conversely, the low internalizing αB7-H3 exhibits a diminished EC_{50} value in HeLa cells relative to the parental CPAb.

Finally, an additional assay was conducted to confirm the cytosolic localization of S4xTrastuzumab and S4xInotuzumab in SKBR-3, Ramos, and HeLa cells. For this purpose, eGFP[42] containing an N-terminal triple glycine sequence (G_4S)-eGFP was coupled to the bispecific constructs via a sortase A reaction. The efficiency of the reaction was analyzed by SDS-PAGE (Figure S7), and the coupling rate was determined to be approximately 50%. To generate confocal laser scanning microscope (CLSM) images, each cell was incubated with 1 μM of the antibody-eGFP conjugate for 8 h, then stained with the Hoechst nuclear marker and LysoTracker, and subsequently fixed (Figure 7).

As anticipated, no intracellular fluorescence was observed for the negative controls on SKBR-3 cells, which included cells only and the S4xInotuzumab constructs (Figures 7A, 7F, and 7G). The masked S4xTrastuzumab-eGFP and the bivalent S4-CPAb variant exhibited minimal to no intracellular fluorescence (Figures 7C and S8C), with the exception of one cell in which a punctate fluorescence pattern co-localized with LysoTracker was observed, indicating lysosomal localization. However, in cells incubated with the unmasked S4xTrastuzumab variant (Figures 7B and S8B), a largely homogeneously distributed fluorescence signal was observed. A comparison with the previously published bivalent S4-CPAb demonstrates a diminished fluorescence intensity following MMP-9 cleavage relative to

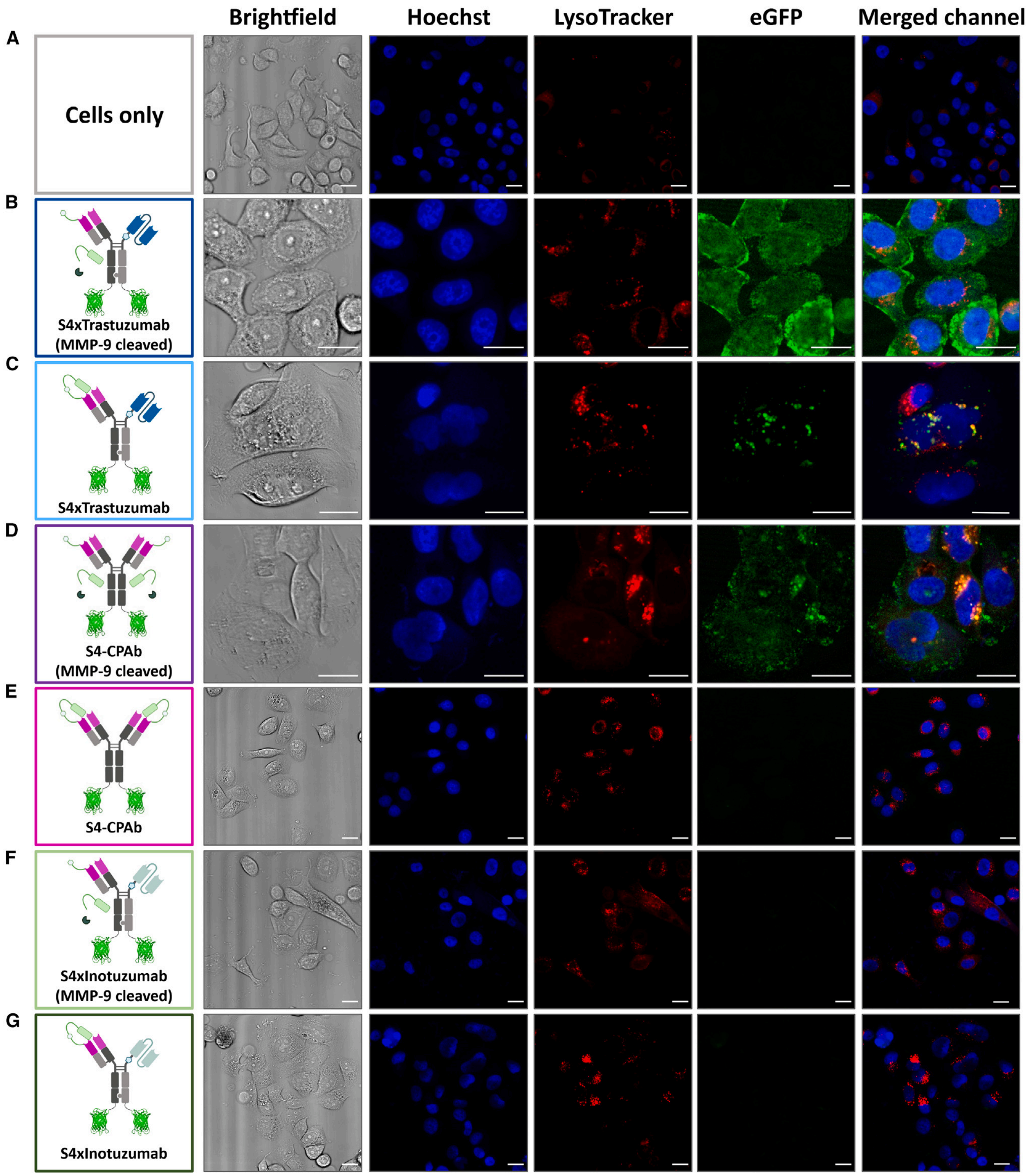

Figure 7. CLSM images of brightfield, Hoechst, or GFP fluorescence channels of SKBR-3 cells treated with 1 μM antibody-GFP conjugates

(A) Without antibody-eGFP conjugate, (B) S4xTrastuzumab-RVRR-eGFP (MMP-9 cleaved), (C) S4xTrastuzumab-RVRR-eGFP, (D) bivalent S4 (MMP-9 cleaved), (E) bivalent S4, (F) S4xInotuzumab-RVRR-eGFP (MMP-9 cleaved), and (G) S4xInotuzumab-RVRR-eGFP were incubated with SKBR-3 cells. The scale bar corresponds to 20 μm. Fluorescence images were generated with ImageJ 1.53c.

demasked S4xTrastuzumab-eGFP (Figures 7D and S8D). However, it also exhibits a homogeneous distribution of eGFP fluorescence within the cell. Nevertheless, co-localization of the native antibody and lysosome is discernible. Punctual bright spots also suggest incomplete cytosol penetration of the antibodies after 8 h. In HeLa cells, which served as a negative control cell line, no intracellular eGFP fluorescence was detected for any of the constructs (Figure S9).

Furthermore, the S4xTrastuzumab and S4xInotuzumab constructs were evaluated in the B-lymphocyte cell line Ramos (Figure S10). As anticipated, the trastuzumab constructs, along with the masked S4xInotuzumab construct, exhibited no intracellular fluorescence signal. However, following MMP-9 cleavage and removal of the mask, a homogeneously distributed intracellular eGFP signal was observed for the S4xInotuzumab-eGFP conjugate, with the exception of the nucleus. This assay, orthogonal to the proliferation assay, additionally enabled the determination of the cell specificity and intracellular localization of the unmasked eGFP conjugates.

DISCUSSION

While the majority of malignant protein-protein interactions occur in the cytosol,[4] methods for therapeutic and diagnostic applications using biomacromolecules remain limited. The current approaches for cytosolic cargo delivery frequently employ either cell-penetrating peptides (CPPs), such as TAT or L17E,[43–45] or cytosol-penetrating antibodies.[7,8,46] As a consequence of the predominantly electrostatic nature of these cell surface interactions eventually leading to cytosol penetration, these approaches frequently result in a non-specific intracellular uptake, which precludes their therapeutic application.[7,43] Moreover, such membranolytic peptides are often quite cytotoxic in themselves, which may preclude the desired specific functional interference of cytosolic-delivered proteins with intracellular targets. A variety of approaches have been pursued to enable tumor cell-specific cargo delivery into the cytosol, including the use of CPP-based endosomal escape peptides (EEPs).[47–49] These consist of a histidine-rich sequence that exhibits reduced cytosol-penetrating capabilities at neutral pH in the extracellular space. In combination with an internalizing tumor antigen-targeting antibody, the EEPs should be taken up intracellularly by receptor-mediated endocytosis. Acidification in the early endosomes leads to protonation of the histidine side chains and thus to the regeneration of membrane lytic activities.[48] An alternative approach, based on cytosol-penetrating antibodies via heparan sulfate proteoglycan binding, relied on reducing HSPG-binding affinity in combination with the coupling of TAA-specific cyclic peptides, targeting tumor-associated integrin $\alpha v\beta 3$ and/or $\alpha v\beta 5$ or tumor-associated epithelial cell adhesion molecule (EpCAM).[13,50,51] A crucial aspect of minimizing non-specificity is the reduction of the number of positively charged amino acid residues, which serves to diminish HSPG-binding affinity and, consequently, non-specific intracellular uptake.[13,48]

In a previous study, we demonstrated that masking the cytosol-penetrating antibody CPAb with a V_L-only domain prevented cytosol penetration in the absence of linker-cleaving MMP-9.[8] However, in the presence of MMP-9, which is often present in the tumor microenvironment, cleavage of the linker can fully regenerate the cytosolic cargo delivery capabilities, resulting in MMP-9-specific cytosol penetration.

In this study, we developed a generic and modular approach to facilitate tumor cell-specific and cytosolic cargo delivery via generation of bispecific antibodies that contain a tumor cell-targeting module. We expected the HSPG-binding and endosomal escape element (RRRRHFDYW) to be located in the CDR3 of the CPAb antibody heavy chain. However, transplantation of the CDR3 onto the scaffold of a VHH domain was insufficient to achieve cytosolic localization (Figures S11–S13). This underscores the necessity for the use of the unmodified CPAb Fab or its masked version. The generation of bispecific format results in a transition from bivalent to monovalent HSPG-binding and endosomal membrane interaction and it remained to be elucidated whether this format still allows for cytosolic localization via endosomal escape. To establish TAA-specific binding, scFvs were utilized in this study that were derived from a B7-H3-binding antibody and the two well-characterized, Food and Drug Administration-approved antibodies trastuzumab and inotuzumab targeting HER2 and CD22, respectively.

On-cell-binding assays demonstrated the tumor cell-specific binding of the bispecific antibody constructs to the respective antigen-overexpressing cell line and the determination of K_D values in the one- to two-digit nanomolar range (Figure 3). These affinities are significantly higher in comparison to the native bivalent HSPG-binding CPAb (K_D[HeLa]: 1 μM and K_D[SKBR-3]: 150 nM; Figure S1C). Hence, in view of low-affinity binding to HSPG, it is not astonishing that the bispecifics displayed cell-specific binding. Interestingly, the presence of the unmasked HSPG-binding module of CPAb had no significant effect on the affinity and maximum binding (E_{max}) (Figure 3) on tumor-target positive cell lines, but resulted in HSPG-dependent binding on tumor-target negative cell lines, confirming the assumption of predominantly scFv-mediated binding, while simultaneously confirming the relevance of the masking moiety in preventing tumor cell-unspecific HSPG interactions.

Following cell binding, efficient endosomal uptake by receptor-mediated endocytosis is a prerequisite for efficient cargo delivery to the cytosol. To investigate this, internalization assays were conducted utilizing a pH-sensitive dye conjugated to the respective constructs, which confirmed the internalization of native trastuzumab and verified that of the bispecific constructs S4xTrastuzumab and S4xInotuzumab (Figure 4). It is noteworthy that comparative internalization assays of the bispecific constructs following the cleavage of the masking unit in those constructs (Figures S2A and S2C) demonstrated a notable reduction in the maximum internalization and AUC values, with an up to 50% decrease. These results confirm the hypothesis that TAA-dependent scFv binding and subsequent internalization occurs primarily and does not involve the CPAb Fab. Additionally, they indicate that the internalized, demasked bispecific antibody undergoes endosomal receptor-release and subsequent endosomal

escape, as evidenced by the increased pH value in the cytosol (pH: 7.0), which significantly reduces the fluorescence intensity of the pH-Dye. It is unlikely that the reduction in E_{max} is due to the intracellular absence of the labeled masking unit, as only two of 62 lysines are located in this region.

As anticipated, the monovalency of antigen binding, exemplified by trastuzumab, leads to a substantial increase in the EC_{50} value, yet a mere half of the normalized AUC in comparison to the bivalent construct. No significant internalization was observed for either the bivalent or monovalent αB7-H3 construct in comparison to the native CPAb after 24 h. Furthermore, no difference was detected when comparing masked and unmasked αB7-H3 bsAb (Figure S2B), which provides additional support for the finding that B7-H3 receptor internalization is generally low resulting in low endosomal accumulation of the αB7-H3 antibody constructs.

We hypothesized that for efficient endosomal escape proteolytic removal of the receptor-binding scFv is required (Figure 1C). Indeed, a construct containing a non-cleavable linker showed almost no cytosolic internalization while introduction of linker containing the recognition sequence for the endosomal enzyme furin (RVRR) resulted in endosomal escape (Figures 5 and S3A). The high binding affinity of scFv to HER2 presumably prevents the release of the construct in the absence of an endosomal cleavable linker, leading to the lysosomal degradation of the antibody-receptor complex. Naturally occurring endosomal protease cleavage sites have been identified in a number of toxins, including *Pseudomonas* exotoxin A and diphtheria toxin.[38,52] The efficacy of both toxins is contingent upon the furin-mediated cleavage of the receptor-binding-domain-receptor-complex from the catalytic domains, which constitutes the entirety of their mechanism of action by cytosolic release.[53,54] Furin is a calcium-dependent serine protease with an active turnover at a broad pH spectrum between pH 5.0 and 8.0.[55] Its natural substrates include transforming growth factor β1 (TGF-β1), pro-albumin, and beta nerve growth factor (beta-NGF).[56–59] The preferred consensus cleavage site is Arg-X-Lys/Arg-Arg, with the sequence Arg-X-X-Arg being the minimum requirement for furin-based cleavage.[38,60] Interestingly, a predominantly lysosomal-cleavable linker containing a legumain recognition sequence (PTN) was also ineffective in delivering PE_{cat} to the cytosol. This may be either due to inefficient cleavage by the enzyme or PE_{cat} degradation in the late endosome/lysosome or both. In the absence of prior unmasking by MMP-9, the S4x-Trastuzumab-RVRR construct only demonstrates a low proliferation inhibitory effect, which can be attributed to the well-known general proliferation inhibition of trastuzumab on SKBR-3 cells[40] and in CLSM imaging co-localization of the antibodies with the lysosomes were observed (Figure 7C). This indicates that the masking moiety still appears to remain bound to CPAb Fab despite decreased pH in the endosomes and lysosomes, resulting in lysosomal degradation. Alpha-fold modeling indicates interactions between the Y59, Y64, N123, and E124, respectively, D66 and D83 from the masking moiety and the R121 and R120 from the HSPG-binding sequence, which is in close proximity to the putative aromatic endosomal escape motive (Figure S14). It is plausible that the ionic interactions between the masking unit and the CDR3 of the CPAb (specifically, R120 with D66 and D83; R121 with E124) should remain intact at lower pH, as the pKa values of the respective side chains (pKa_{Arg}: 12.5; pKa_{Asp}: 3.7; pKa_{Glu}: 4.5) maintain the functional groups within their charged state. The masking of the quadruple-arginine motive may therefore potentially inhibit crucial conformational changes that would otherwise lead to the surface exposure of the endosomal escape motive. Consequently, the masking unit may provide a second safety mechanism to prevent not only HSPG non-specific binding but also general MMP-9-independent endosomal escape.

The significantly disparate EC_{50} values (S4xTrastuzumab: 28.4 nM, S4xInotuzumab: 184 nM, and S4xαB7-H3: n.d.) of the three constructs, in addition to the values of the normalized AUC in the internalization assays facilitate a more comprehensive interpretation of the significance of individual mechanistic steps, particularly in conjunction with subsequent tumor cell-specific cytosol penetration assays in Ramos ($CD22^+$), HeLa ($B7\text{-}H3^+$), and SKBR-3 ($HER2^+$, $B7\text{-}H3^+$) (Figure 6).

The S4xαB7-H3 construct, which demonstrated low internalization, exhibited cell killing only at the maximum concentration of 500 nM. The EC_{50} value for this construct on HeLa cells is approximately 1.6 times higher than that of the parental, bivalent CPAb (200 nM) and 9-times higher than that of the bivalent, unmasked S4-CPAb (55 nM). Nevertheless, the proliferation-inhibiting effect does not appear to result from binding and subsequent internalization of the unmasked CPAb Fab to HSPG, as evidenced by the significantly lower effect observed with the inotuzumab and trastuzumab constructs on HeLa cells as well as the results determined in internalization assays conducted with the unmasked construct (Figure S2). Although the construct does not facilitate a more efficient uptake of the antibody-cargo conjugate by TAA-binding and internalization, a certain degree of cell specificity was observed. Given that the internalization assay is conducted for a shorter period of time (24 h) compared with the proliferation assay, it is possible that slower and less efficient internalization processes may not have been detected, which could otherwise be discernible in the 3-day proliferation assay.

In SKBR-3 cells, the isotype controls demonstrated no cytotoxic effect with or without prior MMP-9 cleavage, further emphasizing the antigen-specific cytosolic penetration of S4xTrastuzumab-RVRR. A proliferation-inhibiting effect was observed in Ramos cells for all unmasked constructs, yet the potencies exhibited significant disparity, resulting in 20- to 40-fold increased EC_{50} values. The general cytotoxicity observed in this assay setup was demonstrated by the combined effect of MMP-9 and the free PE toxin on Ramos cells (Figure S6).

Nevertheless, the EC_{50} values for S4xInotuzumab and S4xTrastuzumab are comparable in the single-digit nanomolar range, representing a significantly higher potency compared with the native CPAb constructs (EC_{50}[Ramos]: n.d. and EC_{50}[SKBR-3]: 42 nM; Figure 6).

The discrepancy in potencies of EC_{50} values from internalization assays and K_D values from binding assays appears to have a negligible effect when the AUC values in the internalization assay are comparable, suggesting a comparable intracellular concentration of the antibody conjugates. These findings, when considered alongside those pertaining to the αB7-H3 conjugates, underscore the crucial role played by the rate of receptor-mediated endocytosis in determining the potency and efficacy of cell-specific cytosol penetration.

It is noteworthy that in some SDS-gels, premature cleavage of the scFvs from the Fc of the asymmetric bispecific constructs having a furin-cleavable linker was observed after production (Figures S4 and S7), which has been previously observed by Geiger et al.[61] However, the proportion of proteins that were cleaved by furin was found to be significantly lower than that of the intact constructs (5% premature cleavage). Since furin is a transmembrane protein that is transported between the cell membrane and the Golgi apparatus and is involved in both intracellular and extracellular cleavage on a variety of cell types,[28] additionally unwanted extracellular cleavage via the target cells could potentially occur. Conducted *in vitro* assays, however, demonstrated high specificity and efficiency in comparison with the bivalent constructs.

The aforementioned constructs serve as a general proof-of-concept for a modular approach to TAA-specific cargo delivery to the cytosol *in vitro*. With regard to potential *in vivo* studies, our hypothesis is that the masking of the construct could serve to minimize off-tumor binding and internalization, as well as prevent endosomal escaping activity of the construct outside the tumor microenvironment. We assume that by enhancing the binding affinity of the constructs to a specific antigen in comparison to the previously published S4-CPAb, the overall specificity as well as antibody concentration in the tumor tissue could be augmented, and potential side effects could be reduced. For potential testing in *in vivo* studies, co-occurrence of the antigen on the tumor and sufficient concentration of MMP-9 in the TME would be required, as well as further linker design for endosomal release while avoiding premature proteolytic cleavage of the TAA-binding antibody fragment in serum. However, due to the masking unit and thus a second safety mechanism, and the diminished HSPG-binding and endosomal escape due to the monovalency, the risk of unspecific cytosol penetration should nevertheless be low. Additional improvements could include the insertion of Fc mutations, specifically LALA respectively P329G LALA, would be advantageous in preventing or inhibiting Fc effector functions, such as antibody-dependent cell-mediated cytotoxicity (ADCC) or antibody-dependent cell-mediated phagocytosis (ADCP)[62] and since the DAR of the bispecific antibodies is approximately 1, the usage of a different coupling strategy resulting in higher DARs could potentially improve the potency of the construct *in vivo*.

We demonstrated a modular approach for bispecific antibodies that facilitate tumor cell-specific cytosolic cargo transport through TAA-specific internalization with additional TME-dependent activation, consequently resulting in two orthogonal safety mechanisms. It is evident that there are numerous potential applications, including the cytosolic delivery of a range of protein-based moieties such as scFvs or nanobodies that bind to intracellular pathogenic proteins (e.g., the oncogenic RAS,[63] BCR-ABL protein,[64] or LMO2[65]) or the delivery of small interfering RNA (siRNA) for the purpose of regulating transcription in cancer cells just to name two. It remains to be elucidated, whether the intracellular concentrations of the cargo-loaded antibodies are sufficient for the induction of a significant, preferably tumor cell-killing effect, through interference with intracellular targets with lower potencies than the here utilized *Pseudomonas* exotoxin A.

MATERIALS AND METHODS

Plasmids

The bispecific constructs were generated using the "knobs-into-holes technique".[18] The CPAb VH was cloned into a pTT5-derived vector via golden gate assembly onto the CH-1 hole-Fc. The variable domains of the tumor-targeting scFvs were linked via a Gly/Ser-based linker (GGGSEGGGSEGGGSEGGG) and fused to the knob Fc. To detect cytosolic penetration, either a tag for sortase A conjugation (LPETGG) or the HiBiT peptide (VSGWRLFKKIS) was inserted C-terminally by genetic fusion. The plasmid sequences were verified by sequencing (Microsynth Seqlab GmbH, Göttingen, Germany).

Information to the sequence, plasmid construction, and protein expression of the masking unit, CPAb, CPAb (118S-121S), (G_4S)-eGFP, and MBP-$(G_4S)_2$-PE_{cat} can be found in Dombrowsky et al.[8]

Cell lines

The adherent cell lines including SKBR-3, HeLa, and HeLa 11ht LgBiT[8] were cultured in Dulbecco's modified Eagle's medium high glucose (Thermo Fisher Scientific, Waltham, MA, USA) supplemented with 10% FBS (Sigma-Aldrich, St. Louis, MO, USA) and 1% penicillin-streptomycin (PS) (Thermo Fisher Scientific) at 37°C and 5% CO_2. Subculturing was performed every 3–4 days. Ramos cells were cultured in RPMI medium supplemented with 20% FBS and 1% PS and subcultured every 2–3 days under humidified conditions.

For protein expression, Expi293F HEK cells were cultured in Expi293 Expression Medium (Thermo Fisher Scientific) at 37°C, 8% CO_2 and 110 rpm. Subculturing was performed every 3–4 days.

Protein expression and purification

Two different protein expression systems were used: bacterial in *E. coli* cells or eukaryotic in HEK Expi293F cells. The bacterial production of G_4S-GFP and $(G_4S)_2$-PE_{cat} was performed in *E. coli* BL21 (DE3). The overnight culture medium, consisting of dYT and the respective antibiotic, was inoculated with the transformed cells. Each 1 L of dYT medium was inoculated with the overnight culture to an OD of 0.1 and allowed to grow to an OD of 0.6–0.8. Protein expression was induced by the addition of 0.5 μM IPTG and the culture was incubated overnight at 25°C and 180 rpm. To purify the constructs, the entire culture was centrifuged, the pellet resuspended in running buffer, and disrupted by sonication. After subsequent

centrifugation and sterile filtration, purification was performed by two-step affinity chromatography using a HisTrap HP column (1 mL, GE Healthcare, Chicago, IL, USA) and a Strep-TactinXT 4Flow column (1 mL, IBA Lifesciences, Göttingen, Germany). eGFP was purified by His-affinity chromatography only.

The expression of bispecific antibody constructs was performed in the HEK Expi293F expression system. ExpiFectamine 293 Transfection Kit (Thermo Fisher Scientific) was used for transient transfection of Expi293F HEK cells according to the manufacturer's protocol. Five days post-transfection, the cell supernatant was sterile-filtered and purified by a two-step purification using the aforementioned HisTrap HP column (1 mL, GE Healthcare, Chicago, IL, USA) followed by a Strep-TactinXT 4Flow column (1 mL, IBA Lifesciences).

All proteins were buffered in PBS (pH 7.4) after purification by either dialysis or a desalting column.

Protein coupling

In order to facilitate protein-protein coupling utilizing sortase A, a penta-mutant variant of sortase A[66] (eSrtA in pET29 [Addgene plasmid: # 75144])[67] was employed. This variant is capable of creating a peptide bond between an engineered C-terminal tag (LPETGG) and an N-terminal triple glycine linker. To obtain the N-terminal glycine, a TEV cleavage site (ENLYFQG) was inserted between the start codon and protein, with cleavage occurring between Q and G. TEV cleavage was performed using an in-house produced SuperTEV protein for 16 h at 24°C. The purification of the proteins from the TEV, as well as from the cleaved linker and MBP, was conducted using Strep-TactinXT 4Flow column (1 mL, IBA Lifesciences). Subsequently, the proteins were rebuffered by dialysis to SrtA buffer (0.05 M Tris, 0.15 M NaCl, 0.005 M $CaCl_2$, pH 7.5). The subsequent SrtA-mediated coupling reaction was conducted in 1× SrtA buffer. In addition to the antibodies and SrtA (0.1 eq.), (G_4S)-eGFP respectively $(G_4S)_2$-PE_{cat} were added to the reaction mixture in an equimolar ratio to the total LPETGG Tag amount. The reaction was supplemented with 2.5 mM (2-hydroxypropyl)-β-cyclodextrin (Sigma-Aldrich) for the reduction of protein aggregation during coupling.[68] The coupling reaction was carried out for 16 h at 24°C and then verified by SDS-PAGE.

Dye-to-antibody coupling and determination of the dye-to-antibody ratio using the pH-sensitive dye pHAb Amine Reactive Dye (Promega, Madison, WI, USA) was performed according to the manufacturer's protocol. After incubation for 1 h at 37°C, excess dye was removed using Zeba Spin Desalting Columns, 7K MWCO (Thermo Fisher Science).

MMP-9 cleavage

For the TME-dependent cleavage of the masking unit of the CPAb construct, an MMP-9 cleavage site (VHMPLGFLGP) was genetically inserted into the linker. The cleavage was performed in sortase A buffer to provide optimal reaction conditions for MMP-9. For pre-activation of recombinant human zinc metalloproteinase (VWR, Avantor, Radnor, PA, USA), the MMP-9 was incubated with 1 mM 4-aminophenylmercuric acetate (APMA) at 37°C overnight. To cleave the masking unit, 0.1 mg of the masked antibody was incubated with 0.1 μg of the pre-activated protease and incubated at 37°C for 24–48 h. Complete cleavage was verified by SDS-PAGE.

Cell binding assay

To ascertain the on-cell affinity of the antibodies on disparate cell lines, 7.5×10^4 cells/well were seeded in a 96-well U-bottom plate. Following a wash step with 0.1% PBS-BSA, the antibodies (in PBS-BSA) were added to the cells at concentrations ranging from 0.01 nM to 2,000 nM, and the cells were incubated on ice for 45 min. Following three washes with PBS-BSA via centrifugation at 4°C and 800 rpm, the cells were treated with the detection antibody, Goat anti-Human IgG Fc eBioscience PE (Invitrogen, Waltham, MA, USA) at a dilution of 1:75 and incubated for a further 30 min at 4°C. After three further washing steps, the cells were analyzed using the CytoFLEX S system (Beckman Coulter, Brea, CA, USA).

Internalization assay

For the determination of antibody internalization by receptor-mediated endocytosis, internalization assays were conducted with the bispecific antibodies, CPAb as well as CPAb (118S-121S). For this purpose, the pHAb Amine Reactive Dye (Promega) was employed in accordance with the instructions provided by the manufacturer. On the day of antibody addition, suspension cells (Ramos) were seeded at a density of 6×10^4 vc/well in RPMI medium supplemented with 10% FBS and 1% PS. Subsequently the cells were treated with antibody-pH-Dye conjugates in a dilution series ranging from 0.15 nM to 500 nM. The cells were incubated for 24 h under humidified conditions, then washed three times with PBS and internalization was analyzed using the CytoFLEX S system (Beckman Coulter).

Adherent cells were seeded 1 day prior to antibody-pH-Dye conjugate treatment with 4×10^4 vc/well each and then the assay was performed in serum-free medium. Further treatment was performed as previously described.

PE-mediated cell proliferation assay

To quantify the cytosol-penetrating properties of the bispecific antibody-PE conjugates in different cell lines, PE-mediated cell proliferation assays were conducted. A colorimetric method utilizing the CellTiter 96 AQueous One Solution Cell Proliferation Assay (Promega) was employed for these analyses. For this purpose, when using adherent cells, 8×10^3 vc/well were seeded into a 96-well flat-bottom plate 1 day prior to the addition of the antibody-conjugate. In the case of Ramos cells, 1.2×10^4 vc/well were seeded. Subsequently, the cells were treated with a dilution series ranging from 0.15 nM to 100 nM, dependent on the cell line, in serum-free DMEM or RPMI 1640 medium (Thermo Fisher Science) with reduced FBS content (10%). Following a 24-h incubation period, the medium was supplemented with 10% FBS and incubated for a further 48 h at 37°C and 5% CO_2 under humidified conditions. Following a 72-h incubation

period, the cells were supplemented with an MTS solution, according to the manufacturer's instructions. The absorbance was then measured at a wavelength of 490 nm utilizing the CLARIOstar Plus microplate reader (BMG LABTECH, Ortenberg, Germany).

NanoBiT assay

The NanoBiT Split-Luciferase Assay (Promega) was used to discriminate between cytosolic and endosomal localization. The previously described cell line, HeLa 11ht LgBiT, was utilized, as well as antibodies that had been genetically fused to the HiBiT peptide at the heavy chain. The intracellular complementation of the truncated luciferase and supplementation with cell-permeable substrates, namely Nano-Glo Vivazine (Promega), permit the monitoring of live cells without the necessity for previous lysis. Twenty-four hours prior to the addition of antibodies, 1.5×10^4 vc/well HeLa 11ht_LgBiT cells were seeded in a 96-well flat-bottom plate and induced by LgBiT expression through the supplementation of 1 μg/mL doxycycline. The cells were treated with the HiBiT-fused antibodies in serum-free DMEM at a concentration of 200 nM and incubated for a further 24 h at 37°C and 5% CO_2 in a humidified incubator. Following a single wash with PBS, the cell-permeable substrate (1x NanoGlo Vivazine) in DMEM supplemented with 10% FBS and 1% PS was added to the treated cells. Following a 2-h incubation period, the luminescence intensity was quantified using the CLARIOstar Plus microplate reader (BMG LABTECH).

Confocal microscopy

To examine the intracellular localization of antibodies, bispecific antibodies were coupled with (G_4S)-eGFP and analyzed using a CLSM. Adherent cells were seeded 24 h prior to treatment at a density of 8×10^4 cells per well in an 18-well μ-slide (ibidi GmbH, Gräfelfing, Germany). The antibodies were added at a concentration of 500 nM in serum-free or serum-reduced medium for 8 h at 37°C and 5% CO_2. Following a two-time PBS rinse, the cells were incubated with 5 μg/mL Hoechst 33342 (H1399; Fisher Scientific, Hampton, NH, USA) and 50 nM LysoTracker Red DND-99 (HY-D1300; MedChemExpress, NJ, USA) in PBS for 10 min at room temperature. To facilitate cell fixation, the cells were first thoroughly washed with PBS and then treated with 4% paraformaldehyde for 20 min at room temperature. Subsequently, the samples were imaged using a Leica TCS SP8 confocal microscope (Leica Microsystems GmbH, Wetzlar, Germany).

Similar procedures were employed for the treatment of suspension cells, although modifications were made to the washing steps. Following the 8-h incubation period, the suspension cells were transferred to an Eppendorf reaction tube. After each wash utilizing PBS or incubation steps with Hoechst, LysoTracker, or 4% PFA, centrifugation was conducted for 3 min at 800 rpm. Subsequently, the supernatant was removed, and the fixed cells were transferred to the 18-well μ-Slide. Finally, the cells were analyzed by CLSM.

DATA AND CODE AVAILABILITY

The authors confirm that the data supporting the findings of this study are available within the article/supplemental information. Further inquiries can be directed to the corresponding author.

ACKNOWLEDGMENTS

We thank Janine Becker for her assistance with antibody production. We acknowledge support by the Bundesministerium für Bildung und Forschung (BMBF—Federal Ministry for Education and Research; Clusters4Future Initiative PROXIDRUGS: ProxiTRAPS [03ZU1109CA]) and the Technical University of Darmstadt's Open Access Publishing Fund.

AUTHOR CONTRIBUTIONS

C.S.D.: Conceptualization, Investigation, Data curation, Writing - original draft. F.K.G.: Investigation, software. D.Z.: Investigation. H.K.: Conceptualization, Project administration, Writing - original draft.

DECLARATION OF INTERESTS

The authors declare no competing interests.

SUPPLEMENTAL INFORMATION

Supplemental information can be found online at https://doi.org/10.1016/j.omton.2024.200931.

REFERENCES

1. Global Burden of Disease Cancer Collaboration, Fitzmaurice, C., Allen, C., Barber, R.M., Barregard, L., Bhutta, Z.A., Brenner, H., Dicker, D.J., Chimed-Orchir, O., and Dandona, R. (2017). Global, Regional, and National Cancer Incidence, Mortality, Years of Life Lost, Years Lived With Disability, and Disability-Adjusted Life-years for 32 Cancer Groups, 1990 to 2015: A Systematic Analysis for the Global Burden of Disease Study. JAMA Oncol. *3*, 524–548.
2. Marschall, A.L.J., Frenzel, A., Schirrmann, T., Schüngel, M., and Dübel, S. (2011). Targeting antibodies to the cytoplasm. mAbs *3*, 3–16.
3. Marschall, A.L.J., Zhang, C., Frenzel, A., Schirrmann, T., Hust, M., Perez, F., and Dübel, S. (2014). Delivery of antibodies to the cytosol: debunking the myths. mAbs *6*, 943–956.
4. Ivanov, A.A., Khuri, F.R., and Fu, H. (2013). Targeting protein-protein interactions as an anticancer strategy. Trends Pharmacol. Sci. *34*, 393–400.
5. Ritchie, M., Tchistiakova, L., and Scott, N. (2013). Implications of receptor-mediated endocytosis and intracellular trafficking dynamics in the development of antibody drug conjugates. mAbs *5*, 13–21.
6. Mayor, S., Presley, J.F., and Maxfield, F.R. (1993). Sorting of membrane components from endosomes and subsequent recycling to the cell surface occurs by a bulk flow process. J. Cell Biol. *121*, 1257–1269.
7. Choi, D.-K., Bae, J., Shin, S.-M., Shin, J.-Y., Kim, S., and Kim, Y.-S. (2014). A general strategy for generating intact, full-length IgG antibodies that penetrate into the cytosol of living cells. mAbs *6*, 1402–1414.
8. Dombrowsky, C.S., Happel, D., Habermann, J., Hofmann, S., Otmi, S., Cohen, B., and Kolmar, H. (2024). A Conditionally Activated Cytosol-Penetrating Antibody for TME-Dependent Intracellular Cargo Delivery. Antibodies *13*, 37.
9. Kim, J.-S., Choi, D.-K., Shin, J.-Y., Shin, S.-M., Park, S.-W., Cho, H.-S., and Kim, Y.-S. (2016). Endosomal acidic pH-induced conformational changes of a cytosol-penetrating antibody mediate endosomal escape. J. Contr. Release *235*, 165–175.
10. Caldwell, E.E., Nadkarni, V.D., Fromm, J.R., Linhardt, R.J., and Weiler, J.M. (1996). Importance of specific amino acids in protein binding sites for heparin and heparan sulfate. Int. J. Biochem. Cell Biol. *28*, 203–216.
11. Hileman, R.E., Fromm, J.R., Weiler, J.M., and Linhardt, R.J. (1998). Glycosaminoglycan-protein interactions: definition of consensus sites in glycosaminoglycan binding proteins. Bioessays *20*, 156–167.
12. Belting, M. (2003). Heparan sulfate proteoglycan as a plasma membrane carrier. Trends Biochem. Sci. *28*, 145–151.
13. Kim, J.-S., Park, J.-Y., Shin, S.-M., Park, S.-W., Jun, S.-Y., Hong, J.-S., Choi, D.-K., and Kim, Y.-S. (2018). Engineering of a tumor cell-specific, cytosol-penetrating antibody with high endosomal escape efficacy. Biochem. Biophys. Res. Commun. *503*, 2510–2516.

14. Masola, V., Bellin, G., Gambaro, G., and Onisto, M. (2018). Heparanase: A Multitasking Protein Involved in Extracellular Matrix (ECM) Remodeling and Intracellular Events. Cells *7*, 236.
15. A. Varki, R.D. Cummings, J.D. Esko, P. Stanley, G.W. Hart, M. Aebi, A.G. Darvill, T. Kinoshita, N.H. Packer, and J.H. Prestegard, et al., eds. (2015). Essentials of Glycobiology (Cold Spring Harbor Laboratory Press).
16. Kim, Y.S., Choi, D.K., Shin, S.M., and Kim, S.H. (2015). Method for positioning, in cytoplasm, antibody having complete immunoglobulin form by penetrating antibody through cell membrane, and use for same. KR20140092673;KR20150103163; WO2015KR07626 *C07K16/30;C07K16/32;G01N33/574*.
17. Yang, H., and Wang, L. (2023). Heparan sulfate proteoglycans in cancer: Pathogenesis and therapeutic potential. Adv. Cancer Res. *157*, 251–291.
18. Ridgway, J.B., Presta, L.G., and Carter, P. (1996). 'Knobs-into-holes' engineering of antibody CH3 domains for heavy chain heterodimerization. Protein Eng. *9*, 617–621.
19. Ulitzka, M., Harwardt, J., Lipinski, B., Tran, H., Hock, B., and Kolmar, H. (2024). Potent Apoptosis Induction by a Novel Trispecific B7-H3xCD16xTIGIT 2+1 Common Light Chain Natural Killer Cell Engager. Molecules *29*, 1140.
20. Lamb, Y.N. (2017). Inotuzumab Ozogamicin: First Global Approval. Drugs *77*, 1603–1610.
21. Yurkiewicz, I.R., Muffly, L., and Liedtke, M. (2018). Inotuzumab ozogamicin: a CD22 mAb-drug conjugate for adult relapsed or refractory B-cell precursor acute lymphoblastic leukemia. Drug Des. Dev. Ther. *12*, 2293–2300.
22. McKeage, K., and Perry, C.M. (2002). Trastuzumab: a review of its use in the treatment of metastatic breast cancer overexpressing HER2. Drugs *62*, 209–243.
23. Vogel, C.L., Cobleigh, M.A., Tripathy, D., Gutheil, J.C., Harris, L.N., Fehrenbacher, L., Slamon, D.J., Murphy, M., Novotny, W.F., Burchmore, M., et al. (2002). Efficacy and safety of trastuzumab as a single agent in first-line treatment of HER2-overexpressing metastatic breast cancer. J. Clin. Oncol. *20*, 719–726.
24. George, B., Kantarjian, H., Jabbour, E., and Jain, N. (2016). Role of inotuzumab ozogamicin in the treatment of relapsed/refractory acute lymphoblastic leukemia. Immunotherapy *8*, 135–143.
25. Thomas, X. (2014). Profile of inotuzumab ozogamicin and its potential in the treatment of acute lymphoblastic leukemia. Blood Lymphatic Cancer *4*, 1–8.
26. Austin, C.D., De Mazière, A.M., Pisacane, P.I., van Dijk, S.M., Eigenbrot, C., Sliwkowski, M.X., Klumperman, J., and Scheller, R.H. (2004). Endocytosis and sorting of ErbB2 and the site of action of cancer therapeutics trastuzumab and geldanamycin. Mol. Biol. Cell *15*, 5268–5282.
27. Cheng, J., Liang, M., Carvalho, M.F., Tigue, N., Faggioni, R., Roskos, L.K., and Vainshtein, I. (2020). Molecular Mechanism of HER2 Rapid Internalization and Redirected Trafficking Induced by Anti-HER2 Biparatopic Antibody. Antibodies *9*, 49.
28. Thomas, G. (2002). Furin at the cutting edge: from protein traffic to embryogenesis and disease. Nat. Rev. Mol. Cell Biol. *3*, 753–766.
29. Dall, E., and Brandstetter, H. (2016). Structure and function of legumain in health and disease. Biochimie *122*, 126–150.
30. Lunde, N.N., Bosnjak, T., Solberg, R., and Johansen, H.T. (2019). Mammalian legumain - A lysosomal cysteine protease with extracellular functions? Biochimie *166*, 77–83.
31. Subik, K., Lee, J.-F., Baxter, L., Strzepek, T., Costello, D., Crowley, P., Xing, L., Hung, M.-C., Bonfiglio, T., Hicks, D.G., and Tang, P. (2010). The Expression Patterns of ER, PR, HER2, CK5/6, EGFR, Ki-67 and AR by Immunohistochemical Analysis in Breast Cancer Cell Lines. Breast Cancer *4*, 35–41.
32. Nejatollahi, F., Jaberipour, M., and Asgharpour, M. (2014). Triple blockade of HER2 by a cocktail of anti-HER2 scFv antibodies induces high antiproliferative effects in breast cancer cells. Tumour Biol. *35*, 7887–7895.
33. Karlsson, M., Zhang, C., Méar, L., Zhong, W., Digre, A., Katona, B., Sjöstedt, E., Butler, L., Odeberg, J., Dusart, P., et al. (2021). A single-cell type transcriptomics map of human tissues. Sci. Adv. *7*, eabh2169.
34. The human protein atlas (2024). Cell line - CD276 - The Human Protein Atlas. https://www.proteinatlas.org/ENSG00000103855-CD276/cell+line.
35. Dijoseph, J.F., Dougher, M.M., Armellino, D.C., Evans, D.Y., and Damle, N.K. (2007). Therapeutic potential of CD22-specific antibody-targeted chemotherapy using inotuzumab ozogamicin (CMC-544) for the treatment of acute lymphoblastic leukemia. Leukemia *21*, 2240–2245.
36. Nath, N., Godat, B., Zimprich, C., Dwight, S.J., Corona, C., McDougall, M., and Urh, M. (2016). Homogeneous plate based antibody internalization assay using pH sensor fluorescent dye. J. Immunol. Methods *431*, 11–21.
37. Tuve, S., Wang, H., Jacobs, J.D., Yumul, R.C., Smith, D.F., and Lieber, A. (2008). Role of cellular heparan sulfate proteoglycans in infection of human adenovirus serotype 3 and 35. PLoS Pathog. *4*, e1000189.
38. Inocencio, N.M., Moehring, J.M., and Moehring, T.J. (1994). Furin activates Pseudomonas exotoxin A by specific cleavage *in vivo* and *in vitro*. J. Biol. Chem. *269*, 31831–31835.
39. Dall, E., and Brandstetter, H. (2013). Mechanistic and structural studies on legumain explain its zymogenicity, distinct activation pathways, and regulation. Proc. Natl. Acad. Sci. USA *110*, 10940–10945.
40. Kokot, A., Gadakh, S., Saha, I., Gajda, E., Łaźniewski, M., Rakshit, S., Sengupta, K., Mollah, A.F., Denkiewicz, M., Górczak, K., et al. (2024). Unveiling the Molecular Mechanism of Trastuzumab Resistance in SKBR3 and BT474 Cell Lines for HER2 Positive Breast Cancer. Curr. Issues Mol. Biol. *46*, 2713–2740.
41. Dixon, A.S., Schwinn, M.K., Hall, M.P., Zimmerman, K., Otto, P., Lubben, T.H., Butler, B.L., Binkowski, B.F., Machleidt, T., Kirkland, T.A., et al. (2016). NanoLuc Complementation Reporter Optimized for Accurate Measurement of Protein Interactions in Cells. ACS Chem. Biol. *11*, 400–408.
42. Cormack, B.P., Valdivia, R.H., and Falkow, S. (1996). FACS-optimized mutants of the green fluorescent protein (GFP). Gene *173*, 33–38.
43. Copolovici, D.M., Langel, K., Eriste, E., and Langel, Ü. (2014). Cell-penetrating peptides: design, synthesis, and applications. ACS Nano *8*, 1972–1994.
44. Ziegler, A., Nervi, P., Dürrenberger, M., and Seelig, J. (2005). The cationic cell-penetrating peptide CPP(TAT) derived from the HIV-1 protein TAT is rapidly transported into living fibroblasts: optical, biophysical, and metabolic evidence. Biochemistry *44*, 138–148.
45. Akishiba, M., Takeuchi, T., Kawaguchi, Y., Sakamoto, K., Yu, H.-H., Nakase, I., Takatani-Nakase, T., Madani, F., Gräslund, A., and Futaki, S. (2017). Cytosolic antibody delivery by lipid-sensitive endosomolytic peptide. Nat. Chem. *9*, 751–761.
46. Kubota, T., Akatsuka, T., and Kanai, Y. (1986). A monoclonal anti-double stranded DNA antibody from an autoimmune MRL/Mp-lpr/lpr mouse: specificity and idiotype in serum immunoglobulins. Immunol. Lett. *14*, 53–58.
47. Cho, Y.W., Kim, J.-D., and Park, K. (2003). Polycation gene delivery systems: escape from endosomes to cytosol. J. Pharm. Pharmacol. *55*, 721–734.
48. Zhao, Y., Jiang, H., Yu, J., Wang, L., and Du, J. (2023). Engineered Histidine-Rich Peptides Enhance Endosomal Escape for Antibody-Targeted Intracellular Delivery of Functional Proteins. Angew. Chem. Int. Ed. Engl. *62*, e202304692.
49. Brock, D.J., Kondow-McConaghy, H.M., Hager, E.C., and Pellois, J.-P. (2019). Endosomal Escape and Cytosolic Penetration of Macromolecules Mediated by Synthetic Delivery Agents. Bioconjug. Chem. *30*, 293–304.
50. Shin, S.-M., Choi, D.-K., Jung, K., Bae, J., Kim, J.-S., Park, S.-W., Song, K.-H., and Kim, Y.-S. (2017). Antibody targeting intracellular oncogenic Ras mutants exerts anti-tumour effects after systemic administration. Nat. Commun. *8*, 15090.
51. Shin, S.-M., Kim, J.-S., Park, S.-W., Jun, S.-Y., Kweon, H.-J., Choi, D.-K., Lee, D., Cho, Y.B., and Kim, Y.-S. (2020). Direct targeting of oncogenic RAS mutants with a tumor-specific cytosol-penetrating antibody inhibits RAS mutant-driven tumor growth. Sci. Adv. *6*, eaay2174.
52. Tsuneoka, M., Nakayama, K., Hatsuzawa, K., Komada, M., Kitamura, N., and Mekada, E. (1993). Evidence for involvement of furin in cleavage and activation of diphtheria toxin. J. Biol. Chem. *268*, 26461–26465.
53. Chiron, M.F., Fryling, C.M., and FitzGerald, D.J. (1994). Cleavage of pseudomonas exotoxin and diphtheria toxin by a furin-like enzyme prepared from beef liver. J. Biol. Chem. *269*, 18167–18176.
54. Moehring, J.M., Inocencio, N.M., Robertson, B.J., and Moehring, T.J. (1993). Expression of mouse furin in a Chinese hamster cell resistant to Pseudomonas exotoxin A and viruses complements the genetic lesion. J. Biol. Chem. *268*, 2590–2594.

55. Molloy, S.S., Bresnahan, P.A., Leppla, S.H., Klimpel, K.R., and Thomas, G. (1992). Human furin is a calcium-dependent serine endoprotease that recognizes the sequence Arg-X-X-Arg and efficiently cleaves anthrax toxin protective antigen. J. Biol. Chem. *267*, 16396–16402.

56. Dubois, C.M., Blanchette, F., Laprise, M.H., Leduc, R., Grondin, F., and Seidah, N.G. (2001). Evidence that furin is an authentic transforming growth factor-beta1-converting enzyme. Am. J. Pathol. *158*, 305–316.

57. Brennan, S.O., and Nakayama, K. (1994). Furin has the proalbumin substrate specificity and serpin inhibitory properties of an *in situ* hepatic convertase. FEBS Lett. *338*, 147–151.

58. Brennan, S.O., and Nakayama, K. (1994). Cleavage of proalbumin peptides by furin reveals unexpected restrictions at the P2 and P'1 sites. FEBS Lett. *347*, 80–84.

59. Urban, D., Lorenz, J., Meyborg, H., Ghosh, S., Kintscher, U., Kaufmann, J., Fleck, E., Kappert, K., and Stawowy, P. (2013). Proprotein convertase furin enhances survival and migration of vascular smooth muscle cells via processing of pro-nerve growth factor. J. Biochem. *153*, 197–207.

60. Vidricaire, G., Denault, J., and Leduc, R. (1993). Characterization of a secreted form of human furin endoprotease. Biochem. Biophys. Res. Commun. *195*, 1011–1018.

61. Geiger, M., Stubenrauch, K.-G., Sam, J., Richter, W.F., Jordan, G., Eckmann, J., Hage, C., Nicolini, V., Freimoser-Grundschober, A., Ritter, M., et al. (2020). Protease-activation using anti-idiotypic masks enables tumor specificity of a folate receptor 1-T cell bispecific antibody. Nat. Commun. *11*, 3196.

62. Wilkinson, I., Anderson, S., Fry, J., Julien, L.A., Neville, D., Qureshi, O., Watts, G., and Hale, G. (2021). Fc-engineered antibodies with immune effector functions completely abolished. PLoS One *16*, e0260954.

63. Tanaka, T., and Rabbitts, T.H. (2003). Intrabodies based on intracellular capture frameworks that bind the RAS protein with high affinity and impair oncogenic transformation. EMBO J. *22*, 1025–1035.

64. Tse, E., Lobato, M.N., Forster, A., Tanaka, T., Chung, G.T.Y., and Rabbitts, T.H. (2002). Intracellular antibody capture technology: application to selection of intracellular antibodies recognising the BCR-ABL oncogenic protein. J. Mol. Biol. *317*, 85–94.

65. Canning, P., Bataille, C., Bery, N., Milhas, S., Hayes, A., Raynaud, F., Miller, A., and Rabbitts, T. (2021). Competitive SPR using an intracellular anti-LMO2 antibody identifies novel LMO2-interacting compounds. J. Immunol. Methods *494*, 113051.

66. Chen, I., Dorr, B.M., and Liu, D.R. (2011). A general strategy for the evolution of bond-forming enzymes using yeast display. Proc. Natl. Acad. Sci. USA *108*, 11399–11404.

67. Hilberg, V., Avrutina, O., Ebenig, A., Yanakieva, D., Meckel, T., Biesalski, M., and Kolmar, H. (2019). Light-Controlled Chemoenzymatic Immobilization of Proteins towards Engineering of Bioactive Papers. Chemistry *25*, 1746–1751.

68. Serno, T., Carpenter, J.F., Randolph, T.W., and Winter, G. (2010). Inhibition of agitation-induced aggregation of an IgG-antibody by hydroxypropyl-beta-cyclodextrin. J. Pharm. Sci. *99*, 1193–1206.

OMTON, Volume *33*

Supplemental information

Tumor-specific cytosol-penetrating antibodies for antigen- and TME-dependent intracellular cargo delivery

Carolin Sophie Dombrowsky, Felix Klaus Geyer, Diana Zakharchuk, and Harald Kolmar

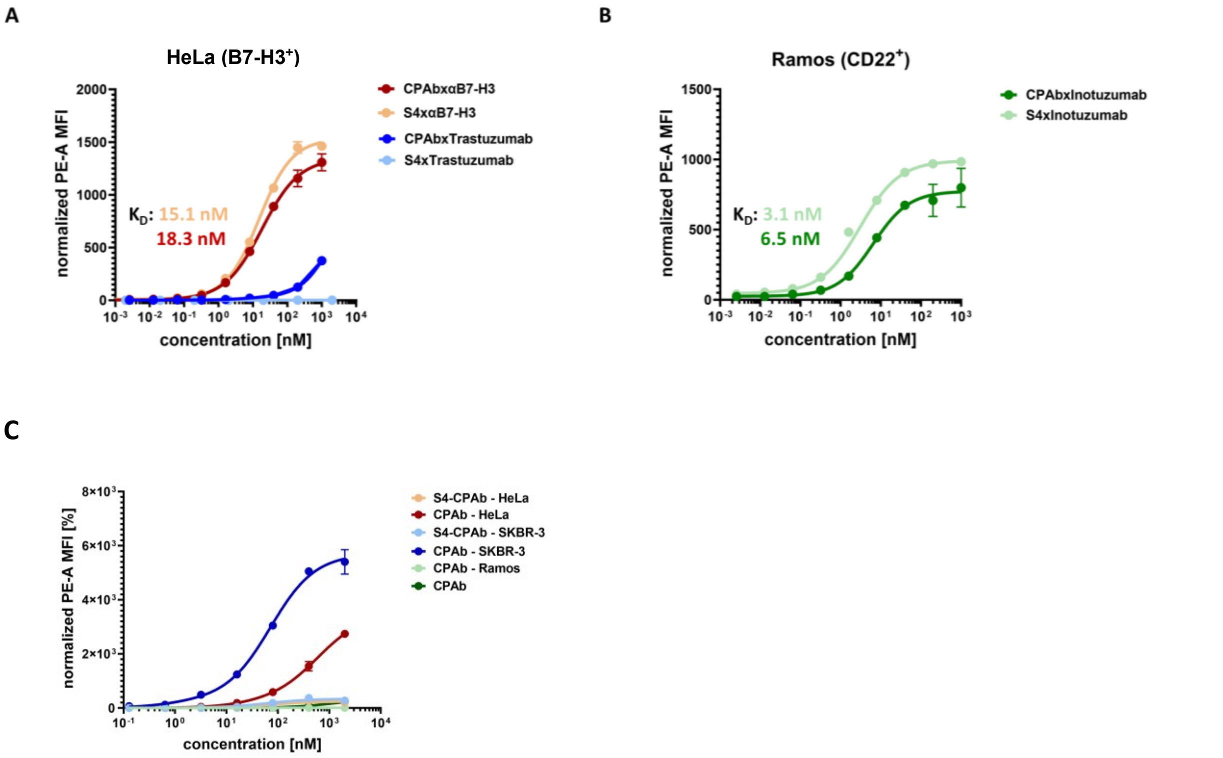

Figure S1. On-cell binding assay of the masked and unmasked bispecific antibodies on Hela and Ramos cells respectively. The cells were treated with concentrations of the compound ranging from 0.002 nM to 2000 nM. The K_D values were calculated from variable slope four-parameter fitting using GraphPad Prism 10.1.0 (316) and presented in the corresponding graph. Comparative analysis for the investigation of HSPG-binding impact on the on-cell binding were conducted in (A) HeLa cells with masked S4xαB7-H3, unmasked CPAbxαB7-H3, and masked and unmasked isotype controls, in (B) Ramos cells with masked and unmasked S4xInotuzumab, and in (C) previously published masked S4-CPAb and unmasked CPAb.

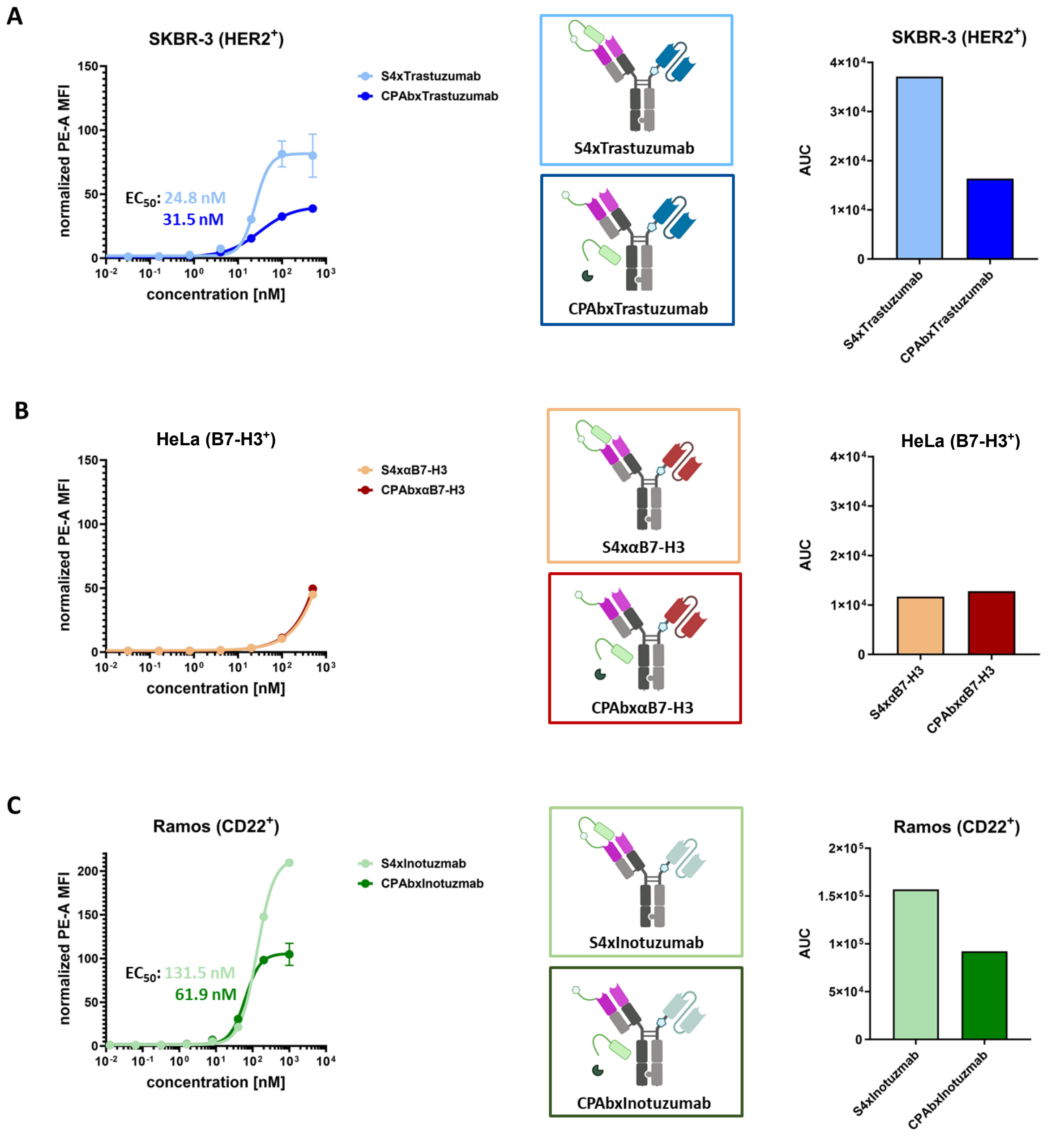

Figure S2. Determination of the internalization of the masked and unmasked bispecific constructs in comparison. The respective cell lines (a) SKBR-3, (b) HeLa, and (c) Ramos were incubated with the antibody-dye conjugates (DAR: S4/CPAbxInotuzumab: 8.9; S4/CPAbxTrastuzumab: 4.6; S4/CPAbxαB7-H3: 8.5). The visualization of the area under the curve of the constructs allows for a comparison of the amount of internalizing antibodies. The resulting data points, depicted as mean and error bars, represent the standard deviation derived from experimental duplicates. The EC_{50} values were determined from a four-parameter variable slope fit using GraphPad Prism 10.1.0 (316).

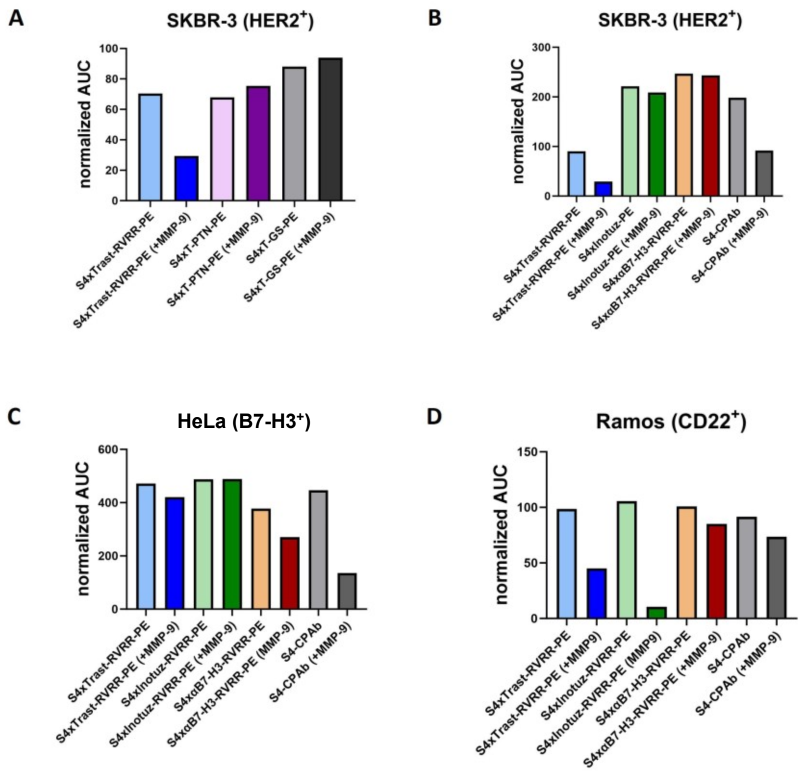

Figure S3. Comparison of linker-dependent and cell-specific cytosolic penetration of bivalent or bispecific constructs in SKBR-3, HeLa and Ramos cells via AUC determination. The visualization of the area under the curve normalized to the upper plateau of the constructs allows for a comparison besides calculated EC_{50} values. The AUCs were determined using GraphPad Prism 10.1.0 (316). (A) Comparison of AUCs of S4xTrastuzumab-RVRR-PE_{cat} (MMP-9 cleaved and untreated), masked and unmasked S4xTrastuzumab-GS-PE_{cat} (non-cleavable) and S4xTrastuzumab-PTN-PE_{cat} (Legumain cleavable) in SKBR-3 cells. AUCs of S4-CPAb, S4xTrastuzumab-RVRR-PE, S4xInotuzumab-RVRR-PE, and S4xαB7-H3-RVRR-PE were compared in (B) SKBR-3, (C) HeLa, and (D) Ramos cells.

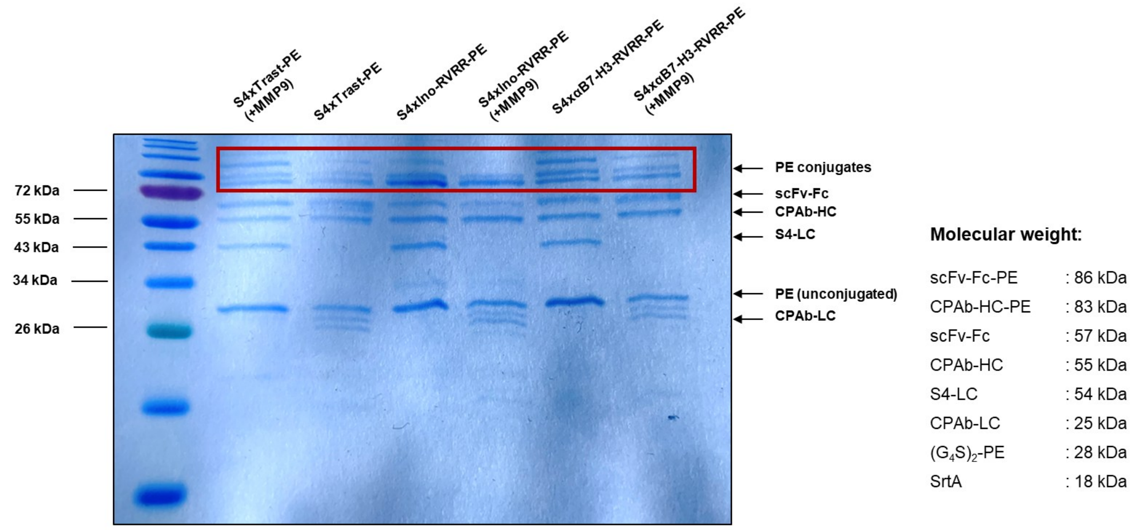

Figure S4. SDS-Gel of antibody-PE_{cat} conjugates. 1.5 µg of S4xTrastuzumab (MMP-9 cleaved and uncleaved), S4xInotuzumab (MMP-9 cleaved and uncleaved), and S4xαB7-H3 (MMP-9 cleaved and uncleaved) were loaded on a reducing SDS-gel. Color Prestained Protein Standard, Broad Range (10–250 kDa) (NEB) was utilized as marker. The red frame highlights the PE_{cat}-coupled heavy chains.

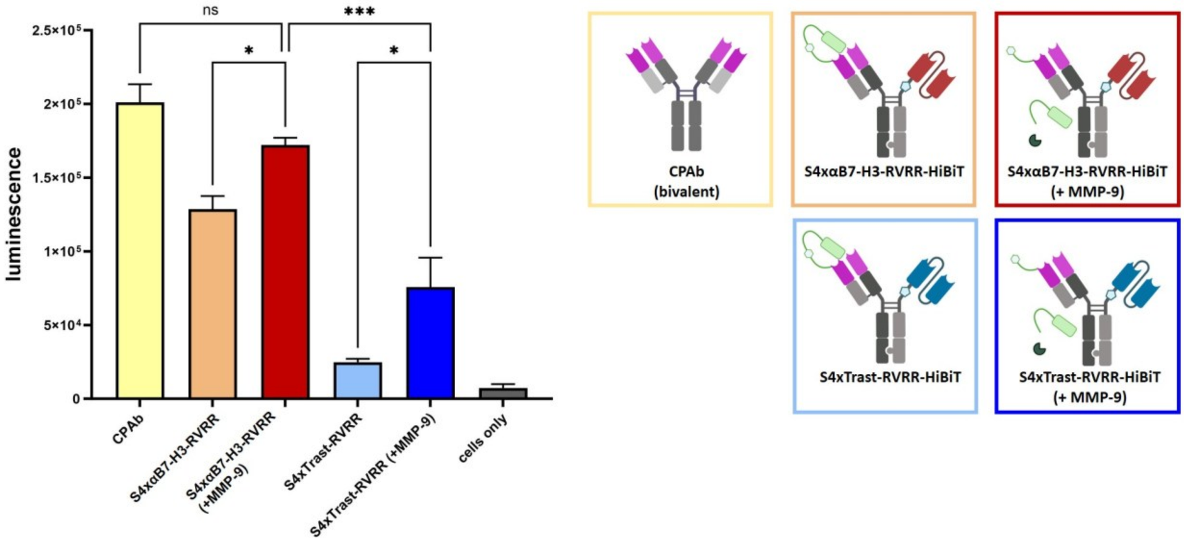

Figure S5. NanoBiT assay analyzing S4xαB7-H3-RVRR-HiBiT variants in comparison with CPAb-HiBiT and S4xTrastuzumab-RVRR-HiBiT in HeLa 11ht LgBiT cells. The results from experimental duplicates were shown as mean values with error bars representing the standard deviation. An unpaired two-tailed t-test (with P value style GP: 0.1234 (ns), 0.0332 (*), and 0.0021 (**)) was utilized to display the significance level (with definition of statistical significance: $P < 0.05$) using GraphPad Prism 10.1.0 (316).

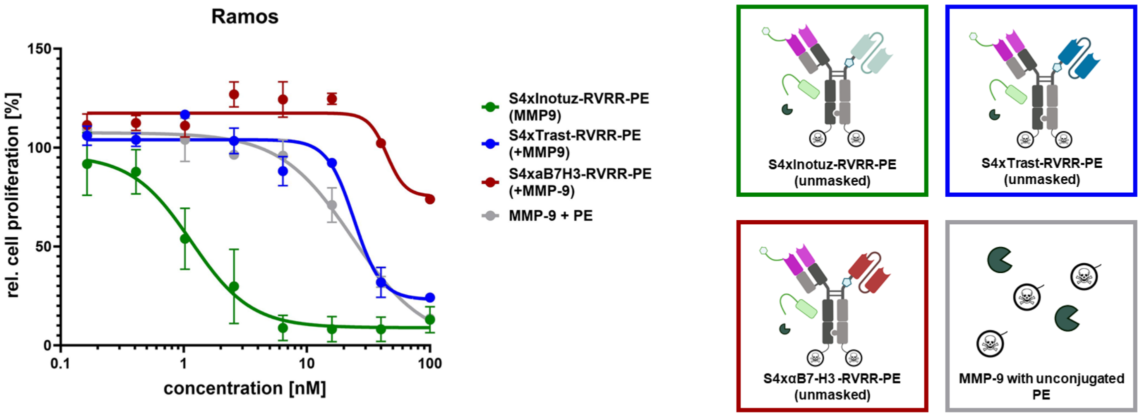

Figure S6. PE-mediated proliferation of MMP-9 cleaved bispecific constructs in Ramos cells in comparison to MMP-9 with free PE_{cat}. The resulting data points are shown as mean and error bars that represent standard deviation derived from experimental duplicates and illustrated using GraphPad Prism 10.1.0 (316).

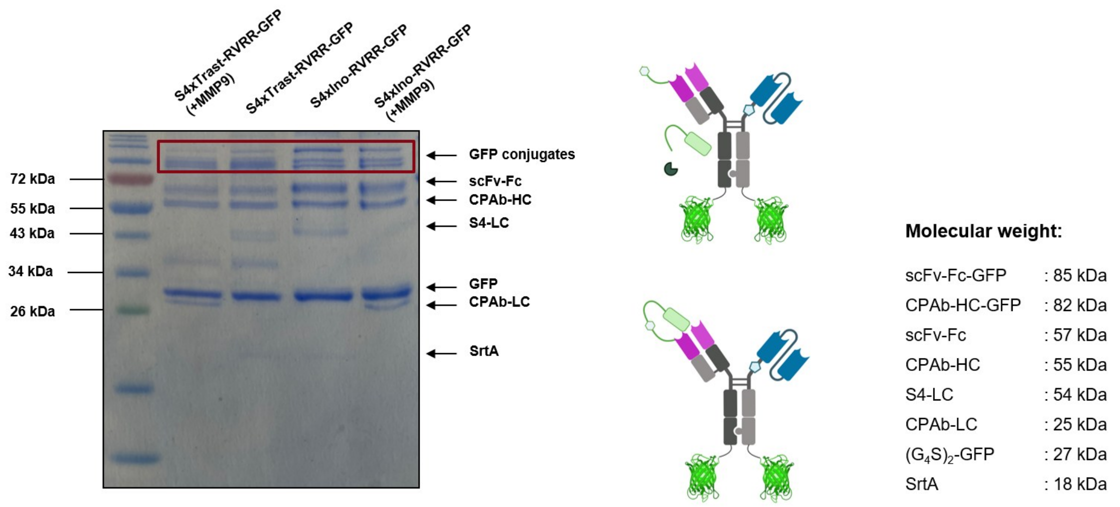

Figure S7. SDS-gel for the verification of the coupling reaction of eGFP with the bispecific constructs. S4xTrastuzumab-RVRR-eGFP and S4xInotuzumab-RVRR-eGFP (MMP-9 cleaved and uncleaved) were loaded on a reducing SDS-gel. The eGFP-coupled heavy chains are highlighted in red. Color Prestained Protein Standard, Broad Range (10–250 kDa) (NEB) was utilized as marker.

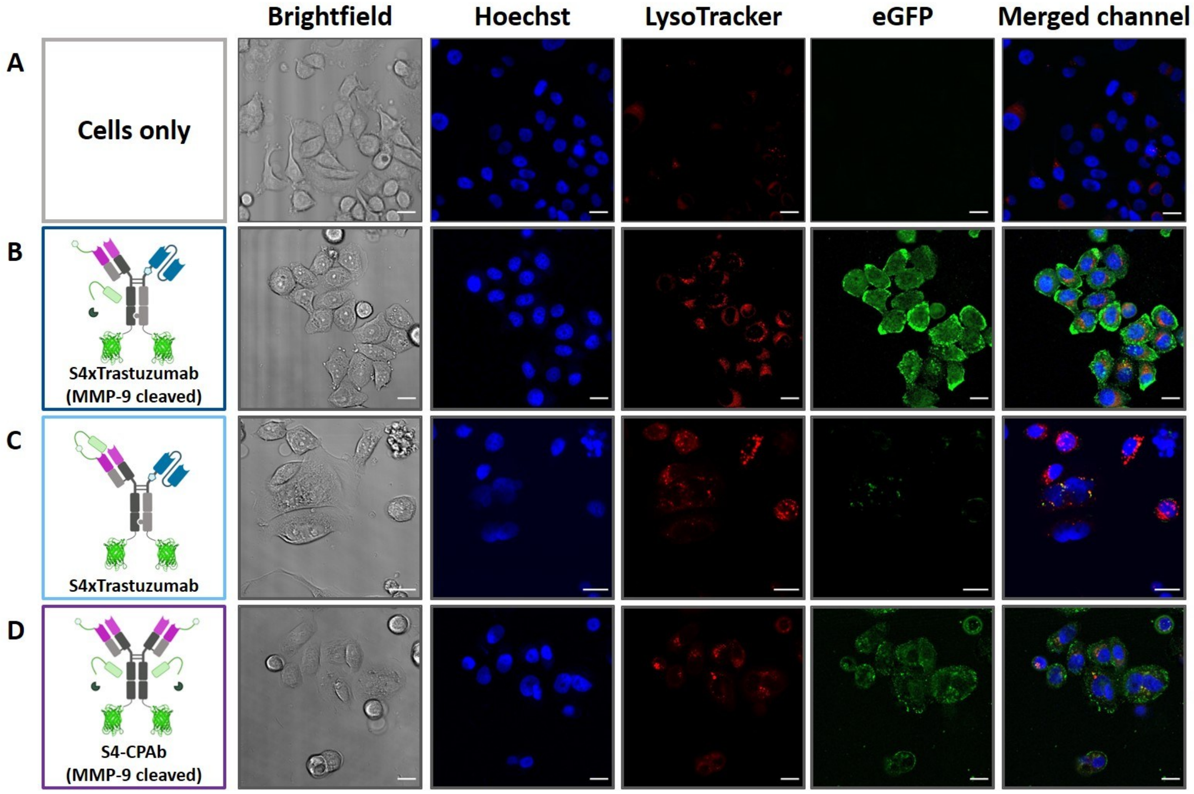

Figure S8. CLSM images of brightfield, Hoechst or GFP fluorescence channels of SKBR-3 cells treated with 1 µM antibody-GFP conjugates. (A) without antibody-eGFP conjugate, (B) S4xTrastuzumab-RVRR-eGFP (MMP-9 cleaved), (C) S4xTrastuzumab-RVRR-eGFP, and (D) bivalent S4 (MMP-9 cleaved) were incubated with SKBR-3 cells. The scale bar corresponds to 20 µm. Fluorescence images were generated with ImageJ 1.53c.

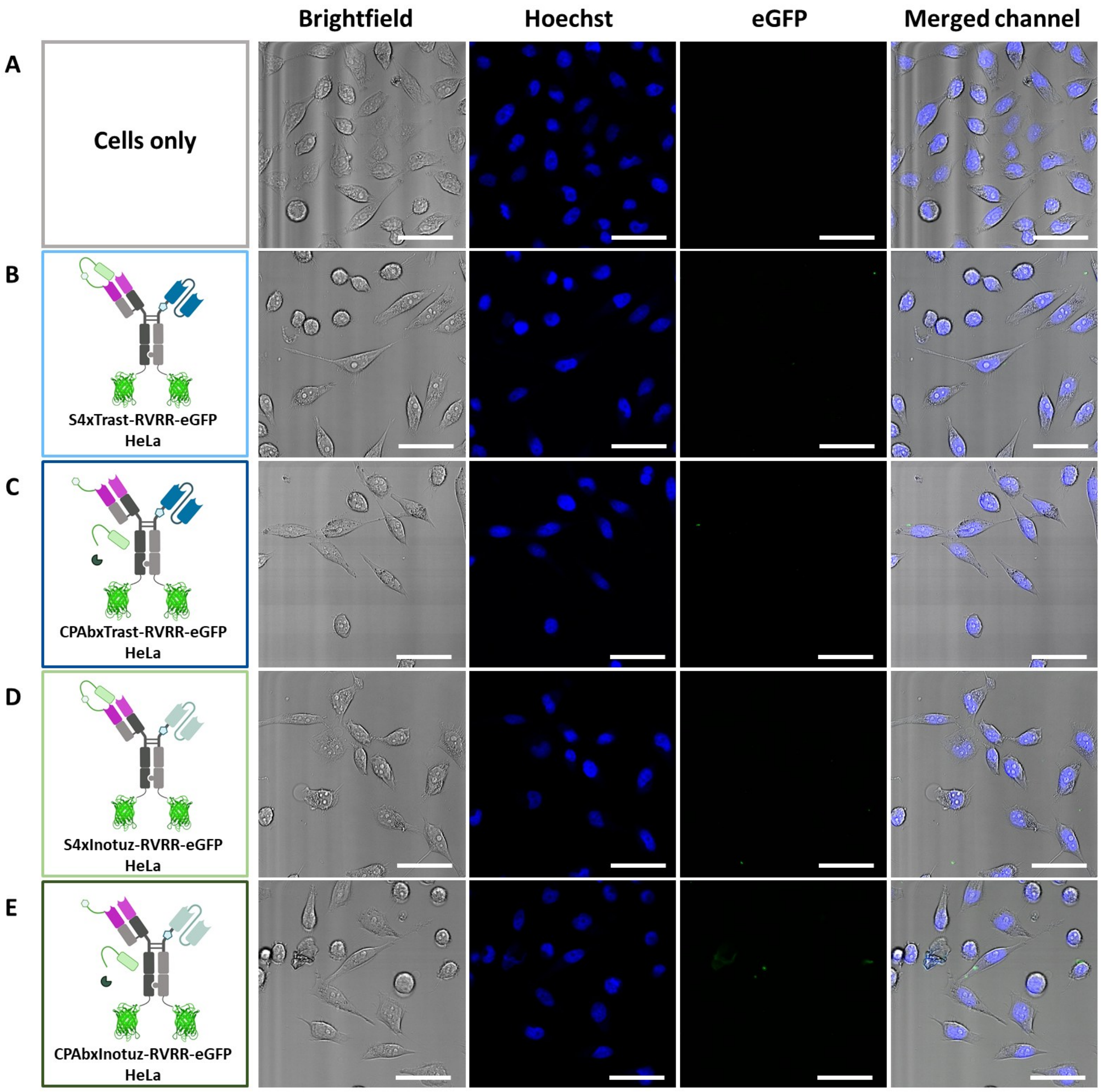

Figure S9: CLSM images of brightfield, Hoechst or GFP fluorescence channels of HeLa cells treated with 500 nM antibody-GFP conjugates. (A) without antibody-eGFP conjugate, (B) S4xTrastuzumab-RVRR-eGFP, (C) S4xTrastuzumab-RVRR-eGFP (MMP-9 cleaved), (D) S4xInotuzumab-RVRR-eGFP, and (E) S4xInotuzumab-RVRR-eGFP (MMP-9 cleaved) were incubated with HeLa. The scale bar corresponds to 50 µm. Fluorescence images were generated with ImageJ 1.53c.

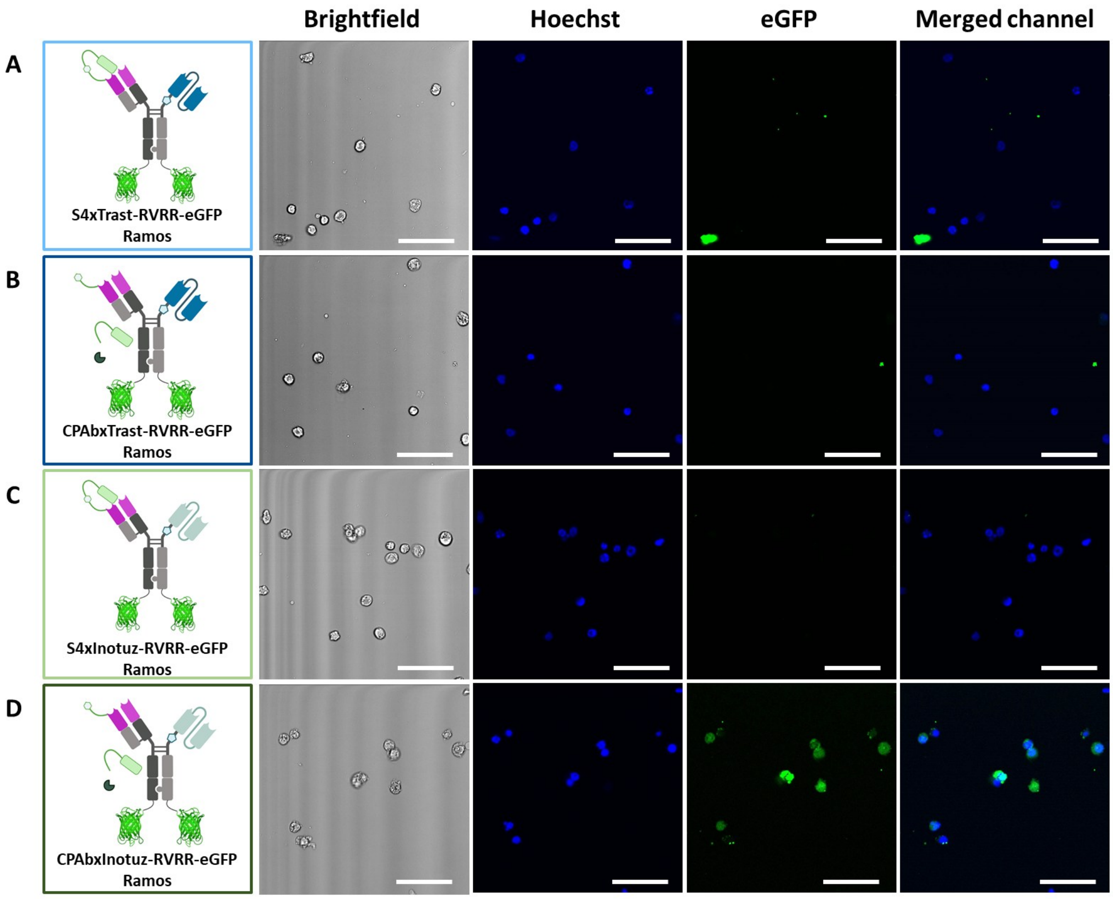

Figure S10. CLSM images of brightfield, Hoechst or GFP fluorescence channels of Ramos cells treated with 500 nM antibody-GFP conjugates. (A) S4xTrastuzumab-RVRR-eGFP, (B) S4xTrastuzumab-RVRR-eGFP (MMP-9 cleaved), (C) S4xInotuzumab-RVRR-eGFP, and (D) S4xInotuzumab-RVRR-eGFP (MMP-9 cleaved) were incubated with Ramos cells. The scale bar corresponds to 50 µm. Fluorescence images were generated with ImageJ 1.53c.

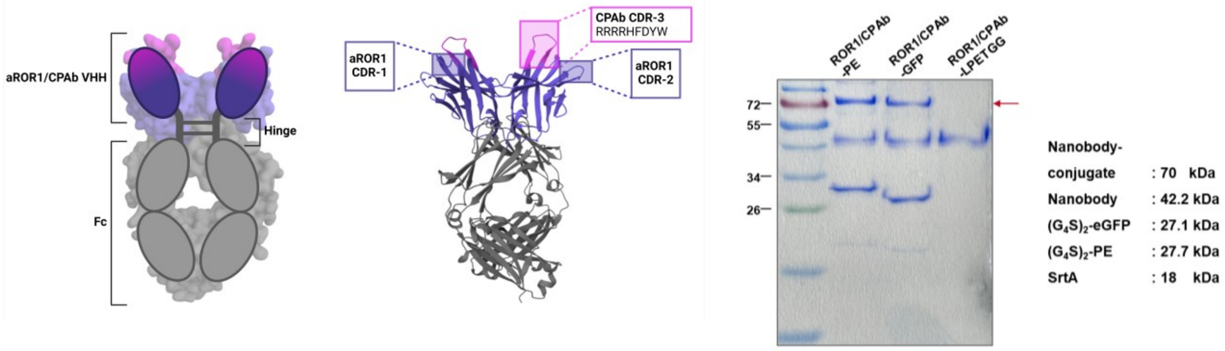

Figure S11. Schematic illustration of the VHH with exchanged CDR3 and SDS-gel of the construct before and after sortase A coupling reaction. Modeling of the VHH-Fc fusion was performed with alpha-fold. The native aROR1 CDR1-2 are highlighted in purple and the exchanged CDR3 in pink. The uncoupled VHH-Fc fusion, the PE_{cat}-respectively eGFP-coupled construct was loaded on a reducing SDS-gel. The coupled heavy chains are highlighted in red.

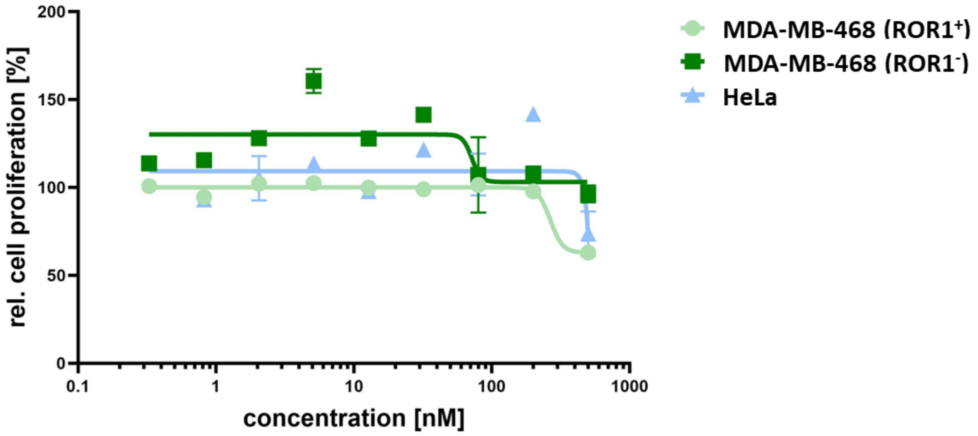

Figure S12. Determination of the cytosol-penetrating capabilities of the aROR1/CPAb VHH-Fc-PE via PE-mediated proliferation assay. The three different cell lines (MDA-MB-468 ROR1$^+$, MDA-MB-468 ROR1$^-$ and HeLa) were incubated with the PE-coupled construct in a dilution. The resulting data points are shown as mean and error bars that represent standard deviation derived from experimental duplicates and illustrated using GraphPad Prism 10.1.0 (316).

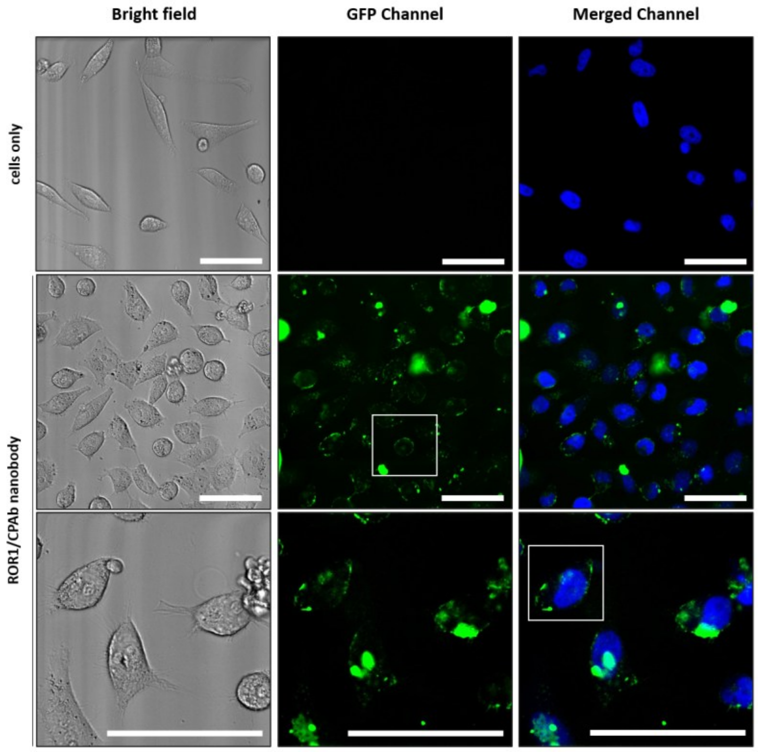

Figure S13. CLSM images of brightfield, Hoechst or GFP fluorescence channels of HeLa cells treated with 1 µM aROR1/CPAb-eGFP conjugate. Non-treated cells served as negative control. The scale bar corresponds to 50 µm. Fluorescence images were generated with ImageJ 1.53c.

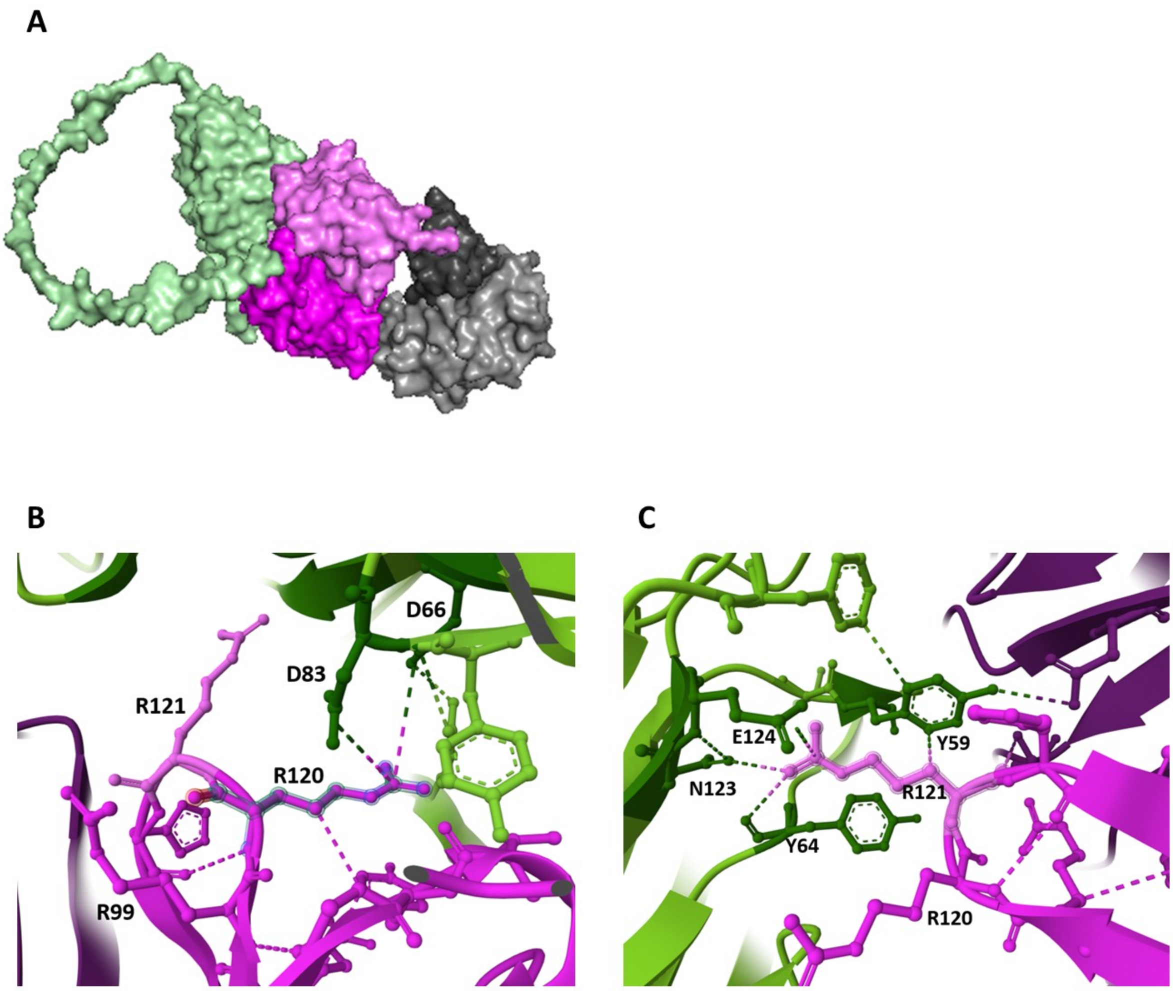

Figure S14. Modeled illustration of the masked CPAb Fab and the potential side chain interactions during the masking process. (A) Alpha-fold proposed modeling of the masking unit to the predominantly CPAb V_L. The masking moiety is shown in green, the CPAb variable domains in pink and the constant regions of the heavy and light chains in grey. (B) Ionic interactions between the Arg120 from the HSPG binding motive with Asp66 and Asp83 from the masking unit. (C) Proposed interactions of the mask with the Arg121 from CPAb V_L based on the alpha-fold model. Illustration via MolStar.
